# Comparing and Evaluating Metagenome Assembly Tools from a Microbiologist’s Perspective - Not Only Size Matters!

**DOI:** 10.1371/journal.pone.0169662

**Published:** 2017-01-18

**Authors:** John Vollmers, Sandra Wiegand, Anne-Kristin Kaster

**Affiliations:** Leibniz Institute DSMZ - German Collection of Microorganisms and Cell Cultures, Braunschweig, Germany; Universidad Miguel Hernandez de Elche, SPAIN

## Abstract

With the constant improvement in cost-efficiency and quality of Next Generation Sequencing technologies, shotgun-sequencing approaches -such as metagenomics- have nowadays become the methods of choice for studying and classifying microorganisms from various habitats. The production of data has dramatically increased over the past years and processing and analysis steps are becoming more and more of a bottleneck. Limiting factors are partly the availability of computational resources, but mainly the bioinformatics expertise in establishing and applying appropriate processing and analysis pipelines. Fortunately, a large diversity of specialized software tools is nowadays available. Nevertheless, choosing the most appropriate methods for answering specific biological questions can be rather challenging, especially for non-bioinformaticians. In order to provide a comprehensive overview and guide for the microbiological scientific community, we assessed the most common and freely available metagenome assembly tools with respect to their output statistics, their sensitivity for low abundant community members and variability in resulting community profiles as well as their ease-of-use. In contrast to the highly anticipated "Critical Assessment of Metagenomic Interpretation" (CAMI) challenge, which uses general mock community-based assembler comparison we here tested assemblers on real Illumina metagenome sequencing data from natural communities of varying complexity sampled from forest soil and algal biofilms. Our observations clearly demonstrate that different assembly tools can prove optimal, depending on the sample type, available computational resources and, most importantly, the specific research goal. In addition, we present detailed descriptions of the underlying principles and pitfalls of publically available assembly tools from a microbiologist’s perspective, and provide guidance regarding the user-friendliness, sensitivity and reliability of the resulting phylogenetic profiles.

## 1. Introduction

It is estimated that over 99% of all environmental microorganisms remain uncultivatable under current laboratory conditions [1,2]. Others grow so slowly, that it can take months or even years to obtain sufficient biomass for genomic analysis [3]. Consequently, classical genome sequencing has been inaccessible for the vast majority of bacterial and archaeal species. In addition to technical challenges in cultivation, it has become increasingly apparent that many model microorganisms that have been cultivated and studied in the laboratory lost important (eco)physiological properties due to adaptive evolution to the applied conditions. The missing genomic data of millions of prokaryotes (“microbial dark matter”) obscures our knowledge of microbial diversity, metabolic potentials and evolutionary histories. Consequently, over the past years an increasing focus has being laid on cultivation-independent methodologies such as metagenomics and single cell genomics approaches to discover and study microorganisms [2,4–6].

In metagenomics not only a single type of microorganism is studied, but the sequence information of the total community within an environmental sample [7]. To this end, numerous cell lysis and extraction protocols, including commercial kits, have been optimized for the efficient, representative and reproducible extraction of environmental DNA [8–11]. Early metagenomic approaches consisted of plasmid- and fosmid-library cloning and subsequent Sanger sequencing [12,13]. However, these methods were time-consuming, costly and required a high amount of input DNA, limiting the sequencing depth to mere glimpses into the most abundant community members. Furthermore, since not all DNA fragments are equally stable within commonly used cloning vectors and hosts, library preparation was highly biased. With the advances of Next Generation Sequencing (NGS) techniques [14,15], the costs for high sequencing depths were significantly reduced (S1 Table) and cloning steps became obsolete. As a result, a much more detailed and less biased view on natural microbial communities was enabled. Apart from facilitating the identification of novel genes and taxa, the increased sequencing depth now also permits the relative quantification of interesting features within a biome based on the coverage of the sequencing reads.

Read lengths of modern sequencing technologies are increasing as well (S1 Table), making a large depth of phylogenetic and community-based functional analyses already possible by directly examining the unassembled sequencing reads. However, the assembly of overlapping reads into continuous or semi-continuous genome fragments–so called contigs or scaffolds—allows an even more detailed view of different aspects within a genomic context. This allows the reconstruction of full-length gene sequences (and even better gene clusters), which can be much more reliably assigned to specific functions or taxa compared to partial gene fragments found on unassembled reads. Longer assembled sequences also enable a more sensitive detection of larger complex genomic features such as Clustered Regularly Interspaced Short Palindromic Repeats (CRISPR), polyketide synthase (PKS) or non-ribosomal peptide synthase (NRPS) gene clusters encoding for secondary metabolites.

In addition, the broader genomic context of interesting features may be further elucidated by sorting (or “binning”) partially assembled genome fragments into categories (so-called “bins”). The aim of this approach is to separate fragments that likely originate from different species while grouping those together that likely belong to the same species, leading to partial or even complete reconstruction of genomes from metagenomic datasets. The range of available metagenomic binning tools is very diverse [16–19] and newer approaches in binning algorithms even allow the sorting of sequence fragments of unassembled reads [20,21], if sufficient read length and quality is provided. The reliability and efficiency of metagenome binning however increases substantially with longer sequence fragments. Therefore, regardless of whether the research goal is to elucidate the taxonomic and metabolic diversity a microbial community, or to attempt the genome reconstruction of individual community members, metagenome assemblies are a crucial step for greatly enhancing subsequent analyses.

The current availability of a vast array of NGS machines (S1 Table) with different throughputs makes metagenome analyses feasible for a larger range of research groups and applications. A good example is the Illumina MiSeq machine, which offers relatively low instrument and run costs in return for a reduced throughput, making this device attractive even for smaller laboratories. As a result, the number and diversity of sequencing projects have risen. This aids in scientific advances while at the same time creating exciting new questions by putting the data generation into the hands of laboratory microbiologists instead of specialized sequencing centers, which–in contrast to the researchers- often have no expertise about the habitats the DNA was sequenced from. Some call this the democratization of sequencing, however, for the handling and analysis of the produced data, a certain extent of bioinformatics expertise is needed. The influence of different methods during distinct stages of metagenomic data generation, processing and analysis has already been discussed in the past [22,23], but the changing properties and peculiarities of sequencing and analysis techniques require constant updates. Unfortunately, available assembly tools are often compared only with respect to assembly-length statistics and genome completeness of highly abundant community members. The overall depth of the represented/recovered community is however, often neglected, especially regarding low abundant community members. Furthermore, most comparisons of assemblers for metagenomics are based on artificial mock communities, which may not always sufficiently represent the peculiarities of real sampling data regarding micro variation and species diversity.

Currently, comparative assessments of metagenome assemblers are found almost exclusively in the original publications of the respective tools. These publications are generally very technical and datasets are often chosen to give a good result with the respective assembler. This easily gives the impression that every single newly available assembly tool is “the best”. For this reason, the "Critical Assessment of Metagenomic Interpretation" challenge (www.cami-challenge.org/) was launched to provide a platform for the comprehensive and systematic comparison of assembly tools and pipelines using standardized “mock community” datasets. Such mock communities are artificial metagenome data sets created by combining known sequencing data of different strains. The results of this initiative will provide a valuable universal benchmark for fundamental assembly characteristics such as contig length statistics and expected mis-assembly rates. Mock community benchmarks give an important overview of the different assembler’s capabilities to reliably reconstruct genomes from high to medium abundant species. However, they provide relatively little conclusions about sensitivity for micro variation and ultra-low abundant community members in actual environmental datasets. Such real data characteristics can be expected to influence the observed phylogenetic and functional diversity when using different assembly tools. Obviously, this also greatly impedes the comparability of publicly available metagenomics datasets processed using different assemblers. Therefore, differences in the observed community profiles produced by different assemblers on real environmental datasets of varying complexity must be taken into account, when processing and analyzing metagenomics data. This requires a certain extent of interdisciplinary knowledge, not only of the underlying microbial ecology, but also of the working principles and peculiarities of the applied processing and assembly tools.

Over the past years a high degree of specialization took place within the scientific community, limiting the analytical resources of individual workgroups: On the one hand, most laboratory microbiologists are unfamiliar with the available bioinformatics methods and their individual pitfalls. On the other hand, trained bioinformaticians are often highly specialized algorithm developers and not directly in touch with laboratory experimental procedures. For this reason, leading members in the omics research field have propagated the promotion of a “bioinformatics middle class” of biologists being competent and informed users, i.e. for “applied” bioinformatics, rather than developers of computational biology tools (e.g. ivory.idyll.org/blog/2015-bioinformatics-middle-class.html).

This publication intends to target this “bioinformatics middle class” by summarizing the basic characteristics of existing computational tools suitable for metagenomics analysis without delving into mathematics and algorithms. A very detailed overview of the underlying principles of sequence assembly is given in S1 Appendix, and a glossary of frequently used assembly terms is given in S2 Appendix **Glossary**. We intend to give an informative overview of freely available assembly tools for metagenomics analysis as well as their applicability for different sample types and research goals from the perspective of a microbiologist. Furthermore, we present and compare the performance of each discussed tool, using real datasets from natural communities of a high diversity forest soil sample and a low diversity algal biofilm sample.

### 1.1 Description and usage of freely available short-read metagenome assemblers

Two major approaches of sequence assembly are commonly used [24,25]: The overlap based approach, as represented by the traditional overlap layout consensus (OLC) method or the more refined string graph, and -more commonly- the de Bruijn graph approach **(for detailed descriptions and explanations please see** S1 and S2 Appendices **Glossary)**. Both approaches use a data structure called a “graph” to represent all connections (edges) between all basic sequence elements, e.g. reads, (called nodes) extracted from the sequence dataset. Such a data structure can be seen as a map, where each basic sequence element (node) represents a location directly connected to a set of neighboring locations via different pathways (edges). Assembly consists of the resolution of this graph by traversing through these connections in such a way, that each element is visited in the correct order, thereby linking them together to form a contiguous sequence (contig). Overlap based approaches are highly suited for the assembly of long sequencing reads, but were found to be too computationally expensive for high throughput short read sequencing data [25]. De Bruijn graph approaches on the other hand enabled the efficient assembly of short read data [26,27]. They have, however, the drawback of splitting each read into subsequences of defined length k (so called k-mers), thereby losing some of the context information inherent in the sequencing reads and making them less suited for long read sequencing technologies.

Currently, most metagenome sequencing projects utilize Illumina sequencing technologies, due to the low DNA input amount requirements and high sequencing throughput. Those reads can be as long as 300 bp. Longer reads >10 kb are produced by e.g. Pacific Biosciences’ SMRT [28], Illumina’s Moleculo [29] or Oxford Nanopore’s MinION [30] technologies (S1 Table) and promise exciting outlooks for future metagenome analysis. However, the lower sequence throughput combined with higher error rates and the requirements regarding the amounts and quality of input DNA remain restrictive factors at the moment. That is why most metagenome assemblers **(**Table 1**)** were designed for handling high throughput short read sequencing data. In the following paragraphs the most commonly used freely available short-read metagenome assemblers are described in the order of their original release dates.

**Table 1:** Overview and basic characteristics of currently used and freely available short read metagenome assemblers. Included are basic characteristics influencing the user friendliness, such as the range of accepted input formats or extent of documentation. The number of total and recent citations indicates the past and present popularity as well as dissemination of the respective tool within the scientific community.

short read assembler	version	last release	Method	input seq format	read pair format	multiple libraries	extensive instructions available	Support	summary of user friendliness	Citations (total/2016)
IDBA-UD	1.1.2	2014	de Bruijn multiple K-mer	.fasta	interleaved only	yes	no	GitHub tickets, email	inflexible, incomplete documentation	481/189
MegaHit	1.0.3	2015	de Bruijn multiple K-mer	.fastq, .fastq.gz, .fasta, .fasta.gz, stdin	interleaved or separate	yes	yes	GitHub tickets, email	simple usage, flexible, well documented	59/39
MetaVelvet	1.2.01	2012	de Bruijn single K-mer	.fastq, .fastq.gz, .fasta, .fasta.gz, .sam, .bam, .stdin	interleaved or seperate	yes	yes (mostly for velvet)	mailing list, email	flexible, well documented	187/72
MetaVelvet-SL	1.0	2015	de Bruijn single K-mer	.fastq, .fastq.gz, .fasta, .fasta.gz, .sam, .bam, .stdin	interleaved or seperate	yes	no	email	Convoluted workflow, flexible	16/11
Ray Meta	2.3.1	2014	de Bruijn single K-mer	.fasta, .fasta.gz, .fastq, .fastq.gz	interleaved or separate	yes	yes	GitHub tickets, email	flexible, well documented	192/73
SOAPdenovo2	2.01	2015	de Bruijn single K-mer	.fastq, fastq.gz, .fasta, fasta.gz, .bam	interleaved or seperate	yes	yes	GitHub tickets, email	well documented	938/334
Omega	1.0.2	2014	String graph prefix + suffix hashtable	.fastq, .fasta	interleaved only	no	yes	email	simple usage, well documented	21/13
metaSPAdes	3.8.0	2016	de Bruijn multiple K-mer	.fastq, .fastq.gz, .fasta, .fasta,gz, .bam	interleaved or seperate	no	Yes	Sourcefourge/GitHub tickets, mailing list, email	flexible, well documented	5/5

#### 1.1.1 SOAPdenovo and SOAPdenovo2

SOAPdenovo is part of the Short Oligonucleotide Analysis Package (SOAP) and was originally developed for single genome assemblies [31]. Nevertheless, it is still commonly used for metagenomes. The assembly process consists of multiple independent steps. First, an optional preassembly error correction step can be performed, in which low abundant k-mers are detected within normally abundant reads. Here, the reads are corrected by substituting the potential erroneous nucleotide positions if the changes are sufficiently supported by the remaining sequencing data (otherwise the reads remain unchanged). Using a de Bruijn graph, the reads are then assembled to contigs which are subsequently scaffolded by iteratively mapping paired end reads back to the graph, beginning with short insert size libraries and continuing with increasing insert sizes, if available. Finally, a “GapCloser” module is used to fill the intra-scaffold gaps based on paired end read information. In 2012 an improved version of SOAPdenovo, called SOAPdenovo2 was released [32]. This version features a more memory efficient and flexible error correction step suitable for larger k-mer lengths. An optional alternative data structure called “sparse de Bruijn graph” adapted from Ye et al [33] was included in addition to the standard de Bruijn graph method. This kind of graph can reduce memory consumption by storing only an evenly distributed subset of actual k-mers, but may produce varying results depending on the amount of allocated memory and computational threads [32]. Therefore, using the “sparse graph” method is only recommended if memory is limited.

Both SOAPdenovo and SOAPdenovo2 are run by a combination of configuration files and command line arguments. The libraries are specified in a configuration file using a simple format, which enables easy automatization with shell scripts. Pre-compiled and ready to use binaries are available from the SOAP webpage (soap.genomics.org.cn), which also contains detailed instructions for running the assembler. Two separate versions of the assembler are included for use with different k-mer lengths. The first version is restricted to short k-mer lengths but has the advantage of requiring less memory, while the second version accepts k-mer lengths up to 128.

#### 1.1.2 IDBA-UD

IDBA (Iterative De Bruijn Graph De Novo Assembler) is a suite of different de Bruijn graph based assemblers, each dedicated for a specific task. Originally, Meta-IDBA was developed for metagenome assembly [34]. This tool attempts to conserve and reconstruct micro variations between closely related sub-strains by partitioning the assembly graph and therefore does not include a preassembly error correction step. However, as stated on the projects home page (i.cs.hku.hk/~alse/hkubrg/projects/metaidba) this component is no longer maintained and the use of IDBA-UD, a tool dedicated to the assembly of datasets with **u**neven read **d**epths, is now recommended for metagenome as well as single cell genome assemblies instead. A basic feature of all IDBA assemblers is the multi k-mer assembly approach, which iterates through a range of k-mer values in order to stepwise improve the de Bruijn graph and the resulting assembly. The process starts at small k-mer lengths and creates preliminary “local assemblies” from contiguous paths within the graph and paired-end read information. After simplifying the graph by removing weakly supported contigs, the remaining local assemblies are used to recover larger k-mers for the next assembly iteration, many of which would not be available if a large k-mer length had been employed directly **(please see** S1 Appendix: **"4.1. Choice of k-mer")**. The coverage information for these recovered k-mers is derived from the coverage information of the corresponding preliminary contig and the mapping reads. Erroneous sequence positions within the reads can be corrected at every iteration, if at least 80% of the mapped reads confirm the correct base type at this position and the read differs from the consensus in no more than 3 positions. In this way, the de Bruijn graph becomes more and more clearly resolved with every iteration step. Unusual for de Bruijn graph based assemblers, IDBA-UD allows even values for the k-mer length *k*. Usually even k-mer lengths are avoided, due to the possible occurrence of palindromes, which introduce branches into the graph that are hard to resolve (please see S1 Appendix: **“2. The de Brujn graph approach”)**. IDBA-UD was originally intended for read lengths of up to 100 bp and k-mer sizes of up to 120. In order to employ higher read lengths and k-mer sizes, some lines within the source code have to be adjusted before compiling and installing the tool. The exact necessary changes are documented in the “Issues” section of the respective GitHub page (github.com/loneknightpy/idba) and can be easily performed without any programming knowledge. However, no software manual is available and most of the command-line usage options are not extensively documented. Another drawback of this tool is the strict limitations of input file formats. Sequences have to be interleaved paired-end reads in a non-compressed sequence file. No other sequence file format than fasta is accepted, which is impractical considering the most common format is fastq. Nonetheless, this tool became widely popular due to its good assembly performance.

#### 1.1.3 MetaSPAdes

SPAdes [35] was originally developed for single cell sequencing data, but has since grown into a veritable program suite for various applications and data types [35–38]. The original SPAdes assembler was designed to address two major issues of single cell sequencing data, namely the uneven read coverage of amplified DNA [2,5] and the necessity to recognize and resolve chimeric sequences. Similar issues also occur in metagenomic datasets, therefore SPAdes should theoretically be applicable for metagenome assemblies as well. However, due to the relatively high memory consumption of this tool, it was not recommended except for low-complex or mini-metagenomes, such as pools of randomly selected single cells [39]. This has changed since the release of SPAdes version 3.7.1, which now includes a dedicated metagenomics assembly pipeline [40] with reduced memory consumption and improved runtimes (http://spades.bioinf.spbau.ru/release3.7.1/).

SPAdes and metaSPAdes are both de Bruijn graph-based, and incorporate several modules which can be used optionally and independently for different processing and assembly steps. By default, Illumina reads are corrected before assembly using BayesHammer [41], a tool which has been designed for sequencing data with highly variable coverage. For the assembly step, SPAdes utilizes an iterative multi-k-mer approach similar to IDBA-UD. But unlike the latter, SPAdes does not reduce the dataset-size by replacing reads with preassembled contigs at each iteration of de Bruijn graph construction. Instead it utilizes the complete read information together with the preassembled contigs at every step. The rationale behind this approach is to be able to account for small indels (insertion or deletions), since they have been observed to occur with higher probability when assembling with short k-mers [35]. For better repeat resolution, SPAdes implements paired de Bruijn graphs. In contrast to most assemblers, which utilize paired end information for simplification steps after standard de Bruijn graph construction, SPAdes directly incorporates this information in the graph by using k-bimers, which are sets of k-mers derived from read pairs and separated by an estimated distance value. SPAdes then iteratively corrects and adjusts the distance estimation of each k-bimer, thereby taking the non-uniform insert length distribution of most shotgun sequencing libraries into account. An important difference is how strain variation is handled in metaSPAdes: micro variations between highly similar “strain-contigs” are combined to form high quality consensus sequences, aiming at the best possible representation of each species instead of every strain variant.

The k-mer range for iterative de Bruijn graph construction is determined automatically based on read length and sequence data type, but can be specified explicitly, using k-mers of up to 128 bp length. SPAdes and metaSPAdes accept a wide range of data types and formats in both compressed and uncompressed form. Datasets may be supplied via the command line or in form of a YAML data set file, which is well documented in the SPAdes manual. A small drawback is posed by the fact that the current version of metaSPAdes does not support multiple input libraries.

#### 1.1.4 MetaVelvet and MetaVelvet-SL

MetaVelvet is an extension of the single genome de Bruijn graph assembler Velvet [42,43]. It makes use of the original Velvet modules velveth and velvetg for k-mer indexing and initial graph building, respectively [42]. Similar to Meta-IDBA [34], MetaVelvet then tries to resolve branched structures within the graph in a way that considers micro variations between related species. By analyzing and comparing the k-mer coverage of each node in the graph, the assembler tries to differentiate between repeats (multiple copies of the same region within the genome of a single species) and chimeras, in order to separate the nodes within corresponding branched graph structures into appropriate sub-graphs. This, however, requires that separate species can be associated with distinct peaks in the node coverage distribution, which may often not be the case for complex communities. An enhanced version of MetaVelvet, called MetaVelvet-SL has recently been published [44]. MetaVelvet-SL utilizes supervised machine learning methods to improve the detection and resolution of chimeric nodes. Here, a rough taxonomic profile has to be generated from the unassembled reads first, e.g. using Metaphlan [45]. Simulated reads are then generated from reference genomes, which are selected based on the taxonomic profiles. The simulated reads are used to create a mock metagenome, which is comparable to the original dataset. A classification module for chimeric nodes is generated from this mock dataset and used in combination with the original sequencing dataset to create a learning module, which in turn is then used to derive the final assembly. Some pre-trained classification models for a few typical environments are already provided (MetaVelvet.dna.bio.keio.ac.jp), but the selection is limited and does not cover the majority of habitats. Incidentally, the predecessor MetaVelvet is currently still being cited considerably more often than MetaVelvet-SL, indicating that it continues to receive far more attention by the scientific community (Table 1), possibly due to the considerably simpler workflow and its broad dissemination.

In order to use MetaVelvet or MetaVelvet-SL, Velvet has to be run on the dataset first. The limits for maximum k-mer length and the number of separate libraries, which may be used, should be adjusted during compilation. Otherwise they are set at restrictively low default values. Reads are accepted in a large variety of input formats and sequence types, and may be compressed in order to save disk space. Average distances between paired-end or mate-pair reads should be explicitly specified. Pre-assembled contigs may be included either as “reference” (used only for scaffolding and graph resolution) or long reads (used for graph building as well as resolution and scaffolding). The use of multiple CPU cores for faster calculation is supported, but not necessarily used at every assembly step.

#### 1.1.5 Ray Meta

Ray Meta is a popular de Bruijn graph-based assembler that was originally developed for single genome assemblies (Ray) [46], but was subsequently adapted to also recognize and handle metagenomics data [47]. Per default, this assembler does not utilize fixed coverage cutoffs. Instead, it analyzes the k-mer coverage distribution in the dataset to determine the minimum coverage value (for which the majority of k-mers can still be expected to be correct), and the average coverage value (displayed by the majority of k-mers) individually for each continuous read path within the de Bruijn graph [46,47]. In the initial de Bruijn graph, a set of high confidence paths (or preliminary contigs) is determined based on k-mer and read coverage and used as seeds for subsequent elongation steps. A “greedy” algorithm is used for elongation, meaning that if two different subsequent paths can extend a seed, only the significantly more confident one is chosen for seed elongation. If none of the possible subsequent paths shows a significantly higher confidence than the other, the seed extension is stopped at this point and each path results in a separate contig. Therefore, any large contigs produced by Ray are highly trustworthy representations of the more abundant genomes in the dataset, but strain variations or homologies between differently abundant community members may lead to an underrepresentation of lower abundant organisms.

Ray parallelizes assembly computations using the Message Passing Interface (MPI) standard. Therefore, a run agent (such as mpirun) of an MPI library such as openMPI (open-mpi.org) needs to be employed [46]. Just like for MetaVelvet, the maximum k-mer length is limited by a value that has to be specified during compilation and installation. The maximum number of separate libraries that may be included in the assembly is fixed at 499, which should be more than sufficient for most cases. The assembler accepts mate-paired, paired-end or single-end libraries given in different file- and compression-formats. Several convenient built-in downstream analysis options, such as the identification and relative quantification of reference species, are also offered.

#### 1.1.6 Omega

Omega [48] is the only example among the here compared assembly tools which is not a de Bruijn graph based assembler. Instead it utilizes the overlap based string graph approach [49] usually used for assembly of long sequencing read data. Nonetheless, this tool has been designed specifically for Illumina sequencing data of metagenomes. Its overlaps are detected using indexed tables of read prefixes/suffixes of defined length for fast and computationally inexpensive handling of high throughput sequencing datasets [50]. By employing the string graph approach [49] it can cope with short Illumina read lengths and the related repeat resolution problems in a similar way as de Bruijn graph approaches, but with the advantage of preserving the complete sequence context information for every read.

Quality control and processing of reads is explicably advised before using this assembler (omega.omicsbio.org/instructions). The prefix/suffix lengths used for identifying overlaps have to be given as command line arguments and influence the sensitivity and specificity of overlap detection. The command line options for assembly are limited but clearly described, making this tool easy to use. Input reads may be supplied in fasta or fastq format, but may not be compressed. The use of multiple processes or threads for speeding up calculations is however not supported, neither is the specification of a memory-usage limit.

#### 1.1.7 Megahit

Megahit [51] was created in the same research group that was involved in the development of SOAPdenovo and SOAPdenovo2 and may be seen as the successor of these tools. It uses a range of k-mer values for iteratively improving assemblies in a strategy adopted from the IDBA assemblers [52]. It employs a new data structure, the "succinct de Bruijn graph" [51], which has been designed to significantly reduce memory requirements. As an additional step to further reduce memory consumption, only k-mers occurring at a frequency above a specified cutoff are retained as “solid-k-mers”, while the rest is removed as potential sequencing errors. By default, the cutoff value is 2, so k-mers occurring at least twice are kept while singleton k-mers are discarded. Because this eliminates not only sequencing errors, but also removes information from genuinely low abundant genome fragments, a “mercy-k-mer” strategy was introduced [51] which recovers discarded k-mers if they provide new and useful information within a trustworthy context: Discarded singleton k-mers that occur on the same read as “solid k-mers” and are needed to connect these “solid k-mers” within the de Bruin graph are recovered and added to the graph. This minimizes loss of sequencing information while still keeping the influence of sequencing errors low.

Megahit accepts single as well as paired-end reads in compressed and uncompressed fasta or fastq format and even piping input data from stdin is supported. The usage is straightforward and well documented and issues can be addressed via an email forum or on the projects GitHub page (https://github.com/voutcn/megahit). Several optional parameter presets may be chosen for different requirements, such as increased sensitivity or the assembly of large and complex metagenomes. Each parameter can also be specified individually. The k-mer range can be set between lengths of 15 and 127. In order to employ higher k-mer values, the source code has to be edited prior to compilation (similar to IDBA-UD, see above), according to instructions, which can be found on the respective GitHub page. Memory usage may be limited to a specified maximum value and adjusted accordingly. Multiple computational threads can be specified and optionally a graphical processing unit can be employed to increase computational power.

#### 1.1.8 Pipelines

Many of the above mentioned tools have been integrated into publicly available pipelines, which combine assembly with preliminary read processing or subsequent analysis steps. Among the most flexible of these pipelines is MetAMOS [53], a modular framework for metagenome assembly, analysis and validation. It can be extended and custom tailored to suit individual needs, but the initial configuration may prove challenging for bioinformatics novices. MOCAT [54] is an alternative but far less flexible pipeline, handling quality trimming, decontamination, assembly, assembly revision and gene prediction. It was released only shortly before SOAPdenovo2 was published and incorporates SOAPdenovo v1.05 and v1.06 as integral assembly components, using optimized parameters and revised error correction as well as scaffolding steps. SLICEMBLER [55] is a pipeline designed for ultra deep sequencing datasets using a “divide and conquer” approach. The read dataset is evenly divided into subsets, which are then assembled independently using an assembler of choice. The assemblies are merged, and reassembled iteratively. The popular metagenome sequence databases IMG/M [56] and MG-RAST [57] have integrated pipelines for metagenome analysis. Many more pipelines are available and new ones are constantly being developed. However, a rudimentary knowledge in Unix bash scripting is sufficient to set up personal pipelines, guaranteeing full control over the entire workflow and allow addressing individual needs and specific biological questions. The required sequence data processing steps are introduced in S1 Appendix. A review of genome analyses tools and a framework for their integration in efficient workflows has been published by Fondi and Liò [58].

## 2. Results and Discussion

**Fig 1:**
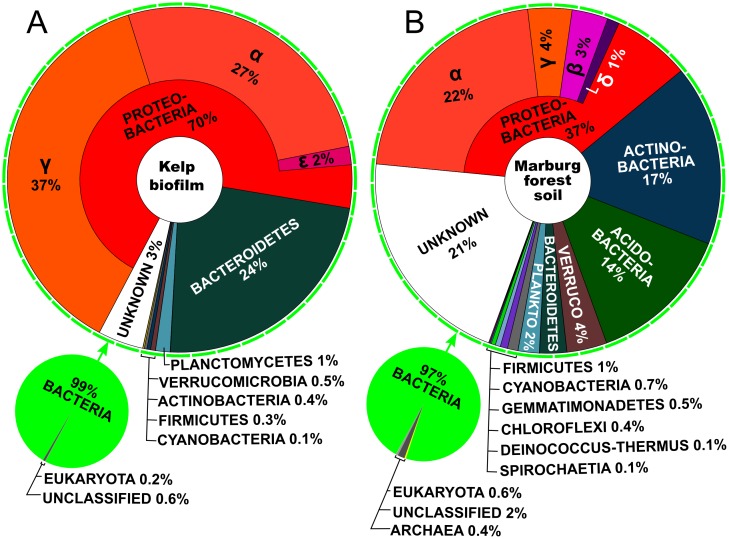
Predicted diversity and relative abundance of organisms represented in the unassembled read datasets. Taxon assignments are based on diamond [68] blastx alignments of all reads against the NCBI nr database and subsequent LCA classification using MEGAN [69]. Large ring charts show detailed breakdowns of the different phyla representing the bacterial fractions in each dataset. The proteobacterial fraction is further broken down into the represented classes of this phylum. Small pie charts indicate the relative abundances of *Bacteria*, *Eukaryota*, *Archaea* and unclassified organisms within the subset of reads which could be assigned at least to the “cellular life form” level. The depicted charts were adapted from visualizations produced by KRONA [70]. Similar analyses, based exclusively on universal marker genes and performed using PhyloSift [71], show almost identical relationships between taxons, albeit with lesser detail and slightly higher estimations for eukaryotic and archaeal fractions (S1 Fig).

The chosen metagenomic samples from Kelp biofilm (KBF) and Marburg forest soil (MFS) (see Materials and Methods) represent bacterial communities of different complexity. Soil communities are usually extremely diverse, sometimes containing millions of bacterial species in only a single gram of soil [59–61]. Biofilms associated with the surface of Kelp, a brown marine macro-algae, are however often dominated by only a few bacterial phyla [62,63]. These predictions were confirmed through taxonomic analyses of the sequencing reads (Fig 1 and S1 Fig). More than 90% of the KBF bacterial community was composed of only two phyla, *Proteobacteria* and *Bacteriodetes*. Already the third most abundant phylum in this dataset, *Planctomycetes*, only makes up 1% of the community, while the remaining phyla are present at ≤0.5% each, indicating a low diversity. These observations are in accordance with previous results by Michelou et al [64], who found mostly *Proteobacteria* and *Bacteroidetes* and a small but persistent fraction of *Planctomycetes* in amplicon based studies of kelp associated microbiomes. In contrast, approximately 80% of the MFS community was composed of up to 12 bacterial phyla, indicating a highly diverse community. The three most dominant phyla in this sample were *Proteobacteria*, *Actinobacteria* and *Acidobacteria* (Fig 1B and S1 Fig), which is in concordance with observations for similar forest soil samples in a study by Baldrian et al [65]. The influence of eukaryotes was found to be minimal in both datasets, making up less than 1% of the total community based on total read alignments (Fig 1) and between 2.7 and 4.4% based on universal marker genes (S1 Fig). The eukaryotic fraction is slightly higher in MFS compared to KBF. Direct comparisons to related datasets within MG-RAST show that eukaryotic abundance was lower than for other comparable soil samples, such as the Rothamsted METASOIL project (project-ID in MG-RAST: mgp405) [66]. To assess how much the differences in overall diversity actually affected the complexity of the MFS and KBF metagenome samples, the read redundancy was determined, based on the median k-mer coverage of each read, using the digital normalization protocol of the khmer suite [67]. The KBF metagenome displayed a high degree of read redundancy, with 32% of the reads originating from high coverage regions (> 20x) and 61% of the reads displaying more than 5x coverage (Table 2). In contrast, the MFS sample showed hardly any read redundancy with only 1% reads originating from high coverage regions and only 6% displaying a coverage above 5x. Hence, the MFS sample represents a highly complex community with relatively low read coverage of individual members, while the opposite is true for KBF, making the assembly of the MFS sample considerably more challenging compared to the KBF sample. This is also reflected in the observed computational costs of the assemblies (S2 Table). Runtime and peak memory consumption for the different assembly runs averaged at 6.4 h and 45 GB for the KBF sample and at 17.7 h and 79 GB for MFS. The only assemblers not showing a distinct increase in RAM usage when assembling the more diverse MFS sample were IDBA-UD and Omega. SOAPdenovo2 achieved the lowest runtime, ranging from 0.8 to 1.8 h using 4 parallel computational threads. Interestingly, the runtime for Omega was also mostly below average, ranging from 2.9–3.6 h. This is remarkable, considering that only one computational thread could be employed. However, the Omega assembly running time increased disproportionally to over 100 h when using an overlap cutoff of 21 for the MFS dataset. The RAM usage of metaSPAdes was close to average for both datasets, but the run times were higher than for most other assemblers. Megahit used the least memory, with a peak RAM usage of 5 GB for the KBF and 12 GB for the MFS assembly. Theoretically this means that both, the KBF and the MFS assemblies could have been performed using Megahit on a standard modern desktop computer with 16 GB or more RAM.

**Table 2:** Basic information on the sequencing read datasets. The total number of reads was determined after adapter clipping and quality trimming. Read redundancy was measured as the fraction of reads occurring at median k-mer coverages above cutoff values of 20x or 5x, respectively, using the khmer suite [67]. KBF: Kelp biofilm sample; MFS: Marburg forest soil sample.

read datasets			read datasets after merging pairs				read redundancy [%]	
Sample	total read number	total read number after quality trimming	merged reads	unmerged paired reads	unpaired reads	median length of merged reads	>20x coverage	>5x coverage
**KBF**	36,119,406	28,409,328	11,495,678	3,189,196	2,228,776	370	32%	61%
**MFS**	33,341,666	28,058,638	11,348,995	2,850,842	2,509,806	360	1%	6%

**Fig 2:**
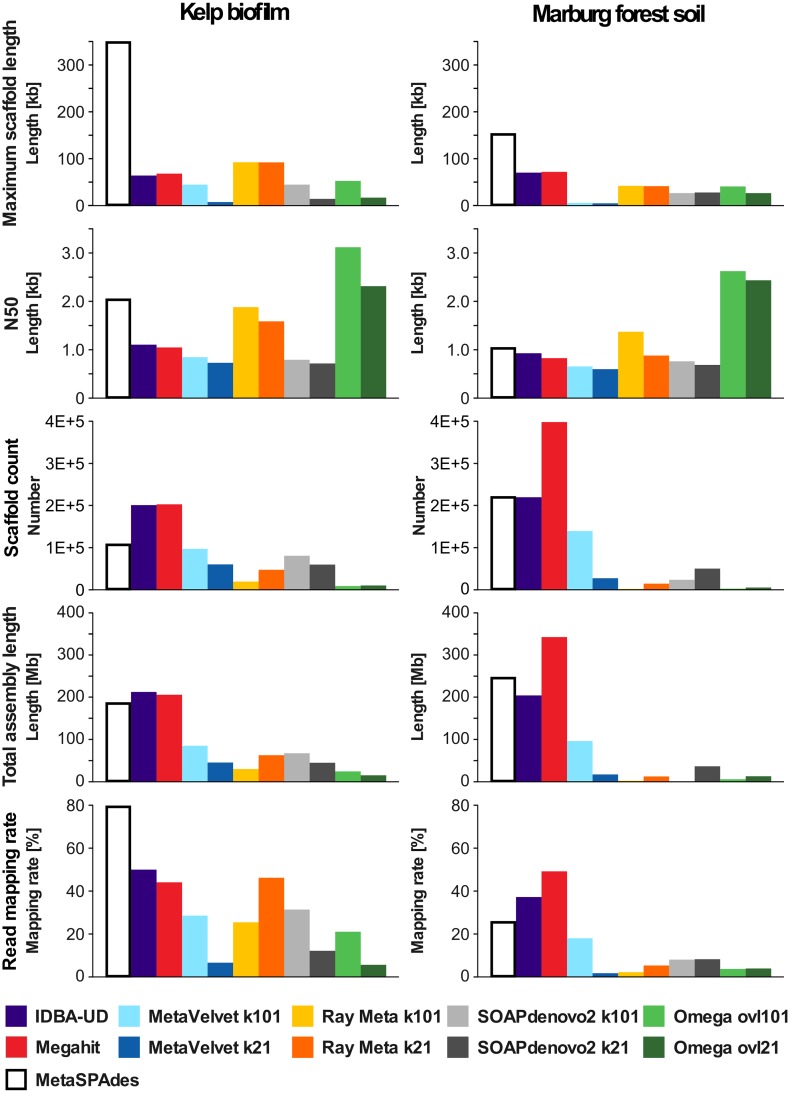
Comparison of individual assembly characteristics produced by different assemblers. Kelp biofilm (KBF) assemblies are shown on the left and Marburg forest soil (MFS) assemblies on the right. Assemblers utilizing single k-mer lengths were tested with two different values for *k*, 21 and 101. All statistics are based on scaffolds larger than 500 bp. The maximum scaffold length indicates the size of the single largest scaffold, while the N50 value represents a weighted average across all scaffolds. The scaffold count represents the total number of scaffolds >500bp. The total assembly length and the read mapping rates indicate how much of the metagenomic sequence information is represented in the assembly. Read mapping rates were determined using the short read mapping tool Bowtie2 and quality-trimmed paired end reads (for a complete list of assembly statistics, please refer to S2 Table).

When comparing the assembly results (Fig 2), drastic differences between assemblers become apparent. The most obvious factors for comparison are simple size statistics, such as maximum scaffold length and N50. For instance, metaSPAdes produced the by far highest maximum scaffold lengths. Furthermore, generally lower scaffold lengths were observed for the complex MFS metagenome in contrast to the less diverse KBF sample, with exceptions for the IDBA-UD and Megahit assemblies. These two assemblers yielded almost equal maximum scaffold lengths for the low diversity KBF and the high diversity MFS sample, illustrating their overall flexibility. However, simple size statistics alone are not sufficient to evaluate the performance of assemblers: Especially for metagenomics, a particularly crucial question is how much of the originally available sequence information was actually incorporated into the assembly, as this directly affects the captured diversity. By evaluating the fraction of unassembled reads, which can be mapped back to the dataset, this question can be quickly assessed in a simple way. Another even simpler, but less informative, way to address this question would be the comparison of the total assembly sizes produced by the different assemblers. Therefore these two factors are also considered in the following assembler comparisons.

The String graph based assembler Omega is a good example for the aforementioned issue. This assembler produced exceptionally high N50 values, indicating a significantly lower proportion of small scaffolds and therefore a less fragmented assembly. This is caused by the fact that for string graph based assemblers scaffold lengths are limited by read lengths and not by k-mer size as it is the case for de Bruijn graph based assemblers. These seemingly good statistics are contrasted, however, by relatively poor read mapping rates and low total assembly sizes, indicating limited information content and therefore low captured diversity in the resulting assembly. For the KBF sample, the assembly results produced by MetaVelvet and SOAPdenovo2 are highly similar to each other, but for the MFS sample, SOAPdenovo2 achieved significantly higher maximum scaffold lengths and N50 values. Nonetheless, this assembler displayed much lower read mapping rates in the MFS assembly than MetaVelvet, indicating that MetaVelvet has incorporated more of the potential sequence information of the original unassembled dataset. This observation is also supported by the higher total assembly length of the MetaVelvet assembly for MFS (Fig 2), illustrating that, for metagenomics, not only size matters, as high scaffold lengths do not necessarily indicate a generally better assembly performance.

**Fig 3:**
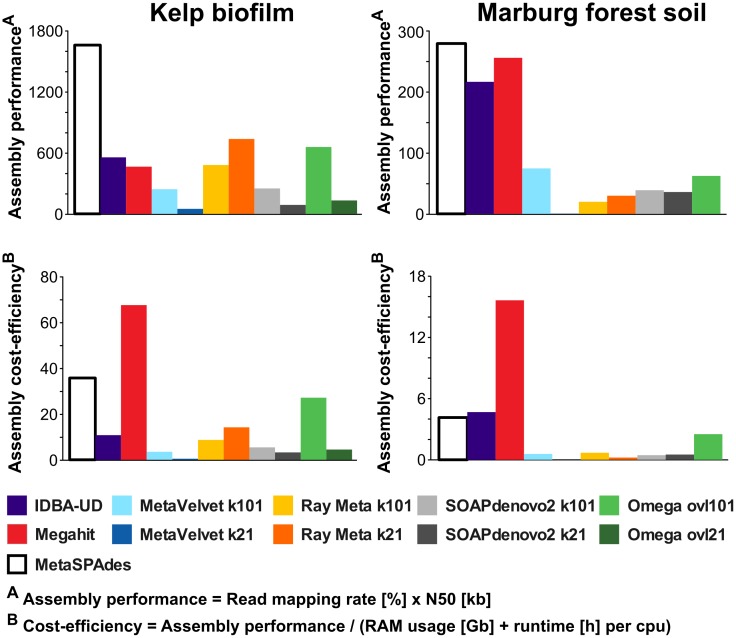
Comparison of assembly performance and cost-efficiency. Kelp biofilm (KBF) assemblies are shown on the left and Marburg forest soil (MFS) assemblies on the right. Assemblers utilizing single k-mer lengths were tested with two different values for *k*, 21 and 101. Assembly performance was defined as the product of the respective read mapping rate (representing information content) and the respective N50. Cost efficiency was defined as the quotient of assembly performance and the sum of RAM usage and runtime per CPU, required for the respective assemblies. For a complete list of assembly statistics, please refer to S2 Table.

**Fig 4:**
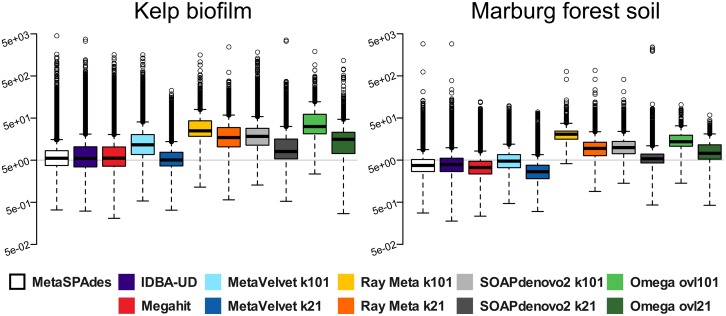
Coverage distribution of assembled scaffolds produced by different assemblers. Kelp biofilm (KBF) assemblies are shown on the left and Marburg forest soil (MFS) assemblies on the right. The boxplots illustrate the relative abundances of scaffolds with different coverage values in the assembled datasets. Coverage values are based on the depth of reads mapping back to the assembled scaffolds >500 bp and were evaluated separately for each assembly and sample using the short read mapping tool Bowtie2 and quality-trimmed paired end reads. A logarithmic scale was chosen for the y-axis in order to account for extreme outliers. For better comparability, grey background lines mark the height of 5x coverage.

In order to improve direct comparability, we therefore defined "assembly performance" as the product of N50 (in kilobases) and read mapping rate (in percent). This way, a good assembly performance is rated not only by average size statistics but also by the included information content. Furthermore, we defined "assembly cost" as the sum of RAM (in Gigabytes) used by the assembler, and the assembly runtime (in hours) per processing core. This allows us to directly rate the "cost-efficiency" of each assembler as the quotient of "assembly performance" and "assembly cost". Direct comparisons of assembly performance and assembly cost-efficiency are shown in Fig 3. The overall best assembly performance for both datasets was achieved by metaSPAdes, followed by IDBA-UD and Megahit (Fig 3, upper bar charts). This indicates a relatively high flexibility of these assemblers and an efficient exploitation of the sequence information represented in read datasets of varying complexity. The highest cost-efficiency (Fig 3 lower bar charts) was by far achieved by Megahit. In the case of metaSPAdes, cost efficiency was still relatively good for the simple KBF dataset, but drastically reduced for MFS. Therefore, computational resources may still become limiting factors when using this assembler on rather large and complex datasets. Ray Meta achieved a high assembly performance on the KBF, but not the MFS dataset. This assembler seems optimized for low complex datasets, such as KBF, which is dominated by only a few bacterial taxa (S1 Fig). Due to the greedy contig extension algorithm implemented by Ray Meta, overrepresented genomes are preferentially assembled into large contigs, while low abundant variants, which may introduce branches to the assembly graph, are more likely to be ignored. Ray Meta produced equally high N50 values for the KBF and the MFS dataset, therefore the low performance of the MFS assembly is caused by reduced read mapping rates (Figs 2 and 3). This is most likely caused by the lower average read coverage of the MFS community members, which raises the question, how well low abundant community members are represented in the low complex KBF dataset. Analysis of the respective scaffold coverage distribution (Fig 4) shows that Ray Meta assemblies are strongly biased towards high coverage genome fragments and display a distinct lack of low coverage scaffolds. This is even more visible in the MFS sample. Generally, a greater bias towards high coverage scaffolds is observed at k101 than at k21, due to the higher likelihood of coverage gaps (please see S1 Appendix **4.1 “Choice of k-mer”**).

The same observation is true for the overlap cutoffs of Omega. In contrast, the metaSPAdes, IDBA-UD and Megahit assemblies display a general tendency to include scaffolds of lower coverage than most other assemblies, with the only exception of MetaVelvet at low k-mer lengths of 21. This indicates a generally higher likelihood for including low abundant community members in the assemblies, and therefore a higher overall sensitivity of these assemblers regarding the represented diversity.

**Fig 5:**
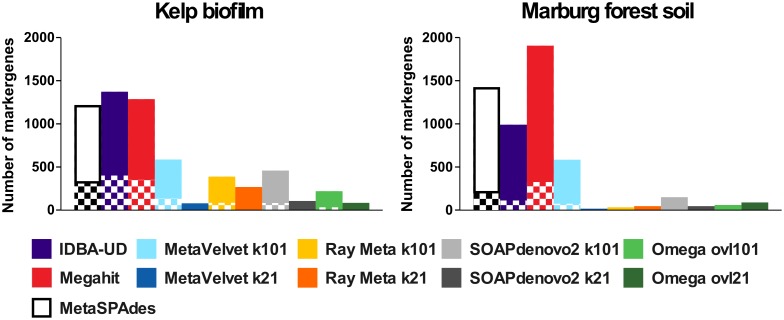
Overall marker gene abundance and redundancy for metagenomes from Kelp biofilm (KBF) and Marburg forest soil (MFS) samples. Bar heights indicate the total abundance of universal bacterial marker gene products >100 amino acids, predicted on scaffolds >500 bp for each assembly, using fetchMG (www.bork.embl.de/software/mOTU/fetchMG.html) [74]. As this value is derived from multiple marker gene types, which co-occur within the same genome, this value does not directly represent the actual number of species captured by the assemblies, rather indicating the overall captured metagenomic potential in terms of species genome completeness as well as diversity. Checkered segments of each bar indicate unique marker gene products, while the solid segments indicate the fraction of closely related and redundant protein sequences, sharing more than 90% amino acid identity. The latter indicate the potential micro diversity captured by each assembler. For a detailed breakup of individual marker gene abundances and diversities please refer to S3 Table.

**Fig 6:**
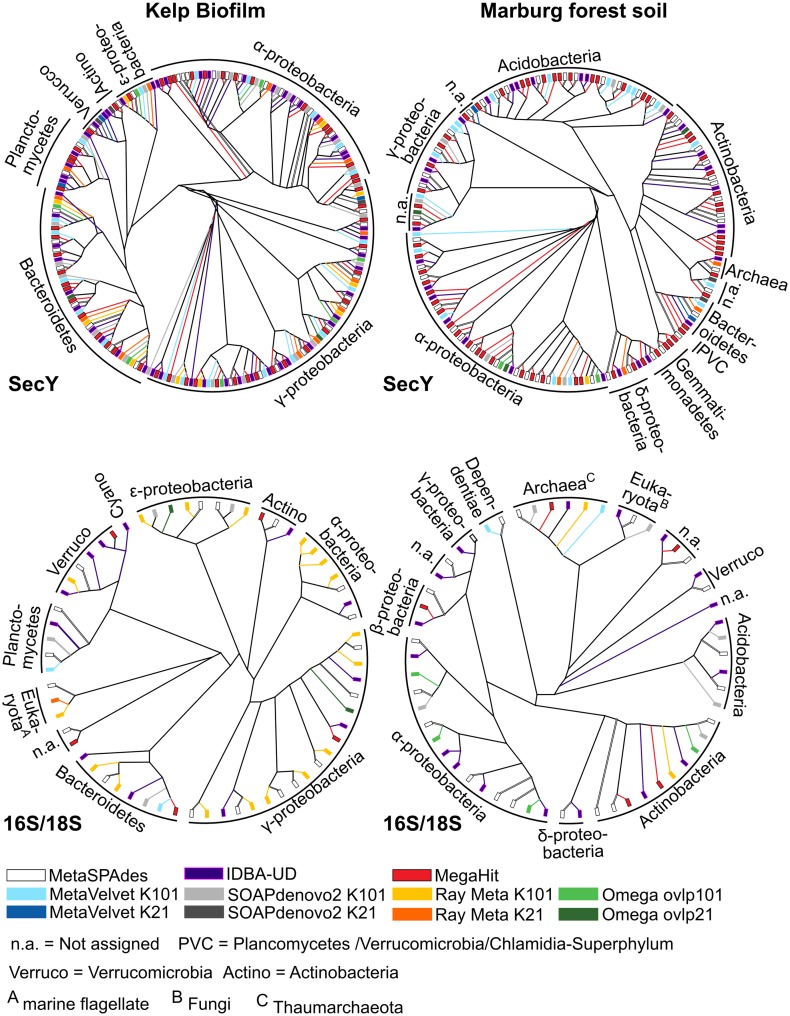
Neighbor-joining phylogeny of abundant marker genes and gene products from Kelp biofilm (KBF) and Marburg forest soil (MFS) samples. Phylogenies are displayed as cladograms, since they provide a more ordered and less-crowded overview than phylograms. Therefore, clustering is shown correctly but terminal branch lengths do not reflect actual phylogenetic distances. **A&B)** Gene product COG201, referring to preprotein translocase subunit SecY. **C&D)** 16S and 18S rRNA gene sequences. Different coloring of terminal branches indicate marker sequences produced by different assemblers according to the color code below the cladogram. Taxonomic classifications are indicated for each observed sequence cluster.

As a result, the total number of universal bacterial marker genes identified in MetaSPAdes, MegaHit and IDBA-UD assemblies is higher than for any other assembler (Fig 5), indicating a higher diversity as well as completeness of represented genomes. A more detailed phylogenetic analysis of pre-protein translocase SecY gene products, which is the most frequently occurring marker gene product in the datasets, (Fig 6A & 6B) confirms this conclusion. The SecY-pyhlotypes produced by metaSPAdes, MegaHit and IDBA-UD cover the largest number of phyla. Even phyla with an estimated abundance below 1% (Fig 1) are included. Examples for such low abundant prokaryotes are *Verrucomicrobia* and *Actinobacteria* for KBF, which are only partially represented by MetaVelvet and are both completely missed by Omega and Ray Meta. This is particularly noteworthy, considering Ray Meta's seemingly good assembly performance for this dataset. Even clearer results are obtained for SecY phylotypes of low abundant *Gemmatimonadetes* in the MFS dataset, which are only represented by metaSPAdes, MegaHit and IDBA-UD. Obviously this can have a drastic impact on various research goals, for instance the reconstruction of metabolic pathways involving low abundant community members at crucial key steps. Interestingly, although every prokaryotic phylum identified on SecY level was represented by all three multi-kmer assemblers, the total number of associated phylotypes was far less for metaSPAdes than for MegaHit or IDBA-UD. This indicates that fewer closely related phylotypes are captured by metaSPAdes, illustrating its relatively low sensitivity for micro diversity.

However, such observations may be influenced by the exact choice of marker genes. On 16S and 18S rRNA gene level, the number of phylotypes recovered from the low diverse KBF metagenome is higher for metaSPAdes and Ray Meta (18 sequences each) than for any other assembler. This indicates that, at relatively high read coverage, metaSPAdes and Ray Meta are both most capable to resolve the highly complex assembly graph structures associated with 16S rRNA genes. It is noteworthy that 16S rRNA genes generally pose a highly challenging problem for metagenome assemblers [72,73]. The main reason for this is the occurrence of multiple highly conserved regions throughout the gene, interspersed by hypervariable sequence stretches. This results in highly complex branched assembly graph structures [72,73]. In theory, a sufficiently high k-mer length, which may span most conserved and hypervariable regions, coupled with a paired end approach should enable the correct resolution of such structures. Nonetheless, the risk of unresolvable structures, or even mis-assemblies, is rather high. Therefore, in many cases the assembly-graph will be split into multiple small contigs, which are mostly confined to the more conserved gene regions. As a result, the number and information content of 16S rRNA gene sequences obtained from metagenome assemblies is often limited. The “consenus” approaches of Ray Meta and metaSPAdes (see the respective descriptions of these assemblers, above) seem to provide an distinct advantage over other assemblers, by enabling the reconstruction of conserved (consensus) gene sequences, despite of complications formed by complex, microvariation-related graph structures. For this reason, assembly results of Ray and metaSPAdes should be considered as consensus sequences, formed by combining sequencing reads which may actually originate from different, though closely related, organisms.

Regardless, none of these sequences were classified as chimeras (see Materials & Methods). Furthermore, in many cases, other assemblers yielded similar sequences with roughly 96% or more sequence identity. Therefore, even though the metaSPAdes and Ray results most likely incorporate the sequence information of multiple different strains, they nonetheless seem to be accurate representations on the genus level. An exception to this assumption is presented by a *Bacteroidetes*-associated sequence obtained by Ray, which contained an obvious misassembly in the form of a ~220 bp insertion. Futhermore, the ability of Ray Meta to efficiently reconstruct small subunit rRNA genes diminished with decreasing read coverage and increasing complexity (Fig 6D). Ray did not represent the low abundant *Actinobacteria* in the KBF sample, and hardly represented any phyla on rRNA level in the highly diverse MFS sample. In the latter case, metaSPAdes and IDBA-UD produced the highest number of phylotypes representing the most phyla.

**Fig 7:**
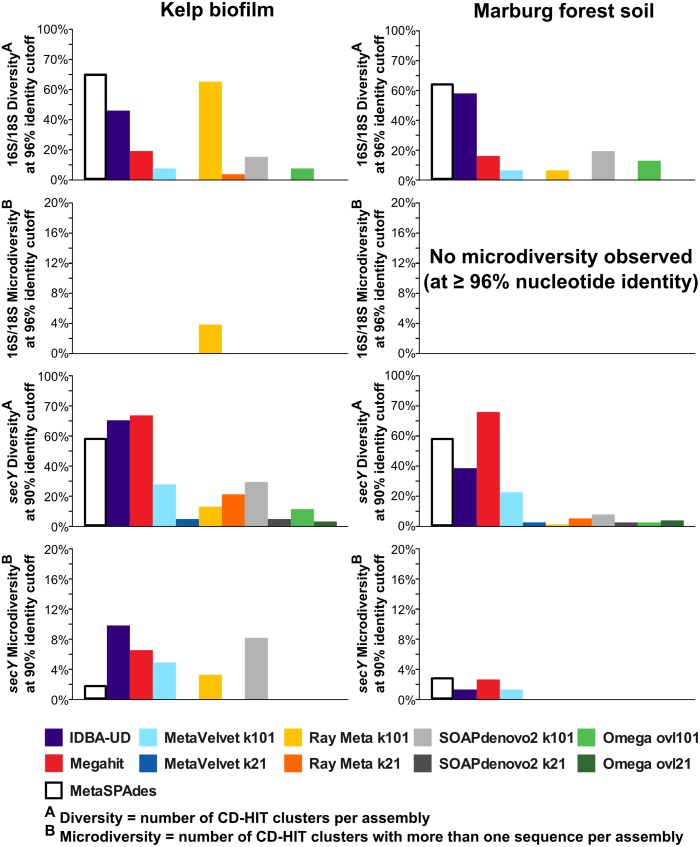
Diversity and micro diversity. Diversity and microdiversity are quantified based on clusterings by CDhit at seqeunce identity cutoffs of 96% (16S/18S rRNA) and 90% (SecY encoding gene sequences). Diversity is represented by the total number of clusters formed for each assembly. Micro diversity is represented by the number of clusters containing multiple sequences originating from the same assembly.

In order to better distinguish and quantify the represented diversity and micro diversity, all detected SecY and 16S/18S rRNA genes were clustered on nucleotide level using cd-hit [75] (Fig 7, S4 Table). The total number of clusters produced by each assembler was counted as diversity, while the number of clusters containing multiple sequences, produced by the same assembler, were counted as micro diversity. Clustering cutoffs were chosen at 96% for rRNA genes and 90% for protein encoding genes. On rRNA gene level, the highest diversity values were achieved by metaSPAdes, IDBA-UD and RAY, at least for the low diverse KBF sample. For the high diverse MFS sample, only metaSPAdes and IDBA-UD yielded particularly high results. However, hardly any micro diversity could be observed, regardless of assembler and dataset, for this marker gene.

Clustering of SecY-encoding genes resulted in similarly high diversity values for metaSPAdes, IDBA-UD and MegaHit, both for KBF and MFS. In contrast, the micro diversity was distinctly lower for metaSPAdes compared to IDBA-UD and MegaHit when analyzing the low diverse KBF sample. However, the difference in micro diversity was far less pronounced for the high diverse MFS sample. This may be explained by the generally lower read coverage in this sample (Table 2), which impedes the ability to detect and reconstruct micro variation for any assembler. Overall, the comparison of observed diversity and micro diversity revealed great differences between the tested assemblers.

**Fig 8:**
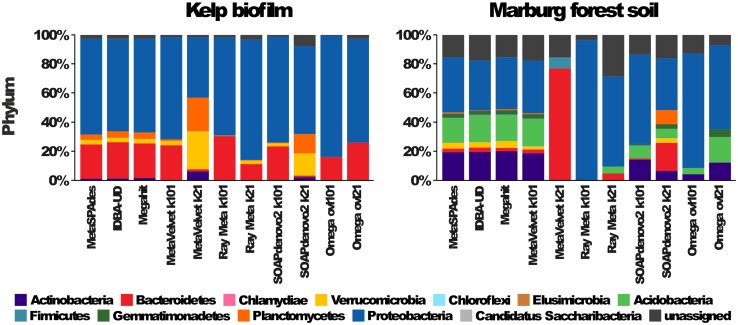
Differences in phylogenetic profiles based on different assemblies of Kelp Biofilm (KBF) and Marburg forest soil (MFS) samples. Phylogenetic profiles are based on 40 single copy marker gene products identified with fetchMG (www.bork.embl.de/software/mOTU/fetchMG.html) and annotations based on alignments against the NCBI-nr database and the least common ancestor (LCA) method implemented by MEGAN5 [69]. The phylogenetic profiles show the number of marker gene products assigned to different taxa on phylum level. Additional profiles showing the class and order levels are given in S2 Fig.

Consequently, annotation and analysis of the complete single copy universal marker genes in each assembly, using Megan [69], resulted in drastically different taxonomic profiles (Fig 8, S2 Fig). As to be expected, assemblies of the high diversity MFS sample generally display more distinct taxa than assemblies of the KBF sample, and a higher amount of "unassigned" sequences. The most complex profiles, based on the number of distinct taxa, were produced by metaSPAdes, IDBA-UD and Megahit closely followed by the k101-assemblies of MetaVelvet and SOAPdenovo2. Overall, the MetaVelvet profiles for k-mer length 101 are remarkably similar to IDBA-UD and Megahit profiles, considering the fact that MetaVelvet assemblies yielded in far less marker genes (Fig 5, S3 Table). However, at k-mer lengths of 21, Metavelvet and SOAPdenovo 2 show a distinct bias towards taxa, which are low abundant in the other assemblies’ profiles (Fig 8, S2 Fig). Interestingly, the opposite is true for Ray Meta assemblies, which seem to exaggerate the proportions of high abundant taxa. Generally, the profiles generated from Ray Meta and Omega assemblies differ the most from all other assemblers and show the least diversity. Therefore, metaSPAdes, Megahit and IDBA-UD assemblies appear to give the most complete view of the taxonomic composition in the different samples.

**Fig 9:**
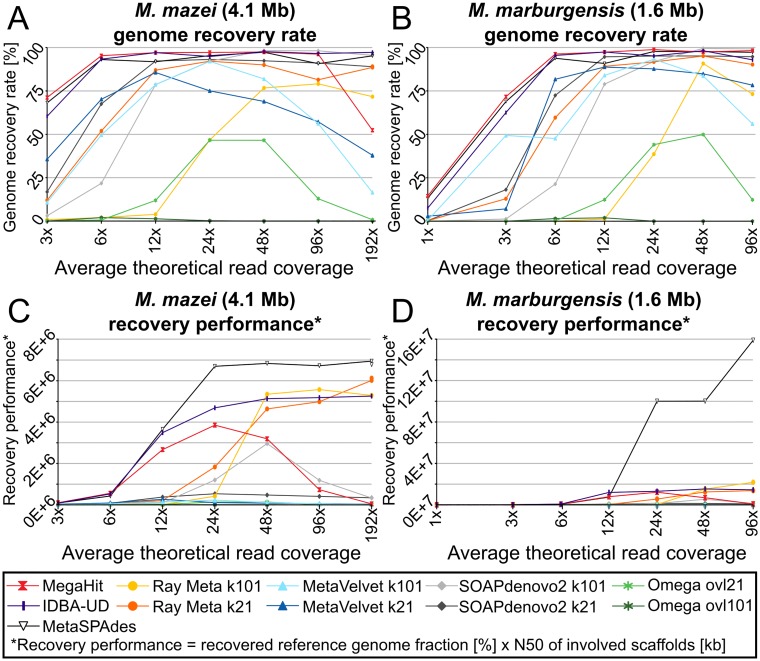
Recovery of known reference genomes from artificial reads spiked into the kelp biofilm dataset. “Genome recovery rates” **(A&B)** are here defined as the fraction (in percent) of each reference genome, which could be reconstructed by each assembler. “Recovery performance” **(C&D)** is here defined analogous to “assembly performance” in Fig 3, as the product of the genome recovery rate and the N50 of the involved scaffolds. A high recovery performance value, indicating a good recovery performance, is therefore achieved by recovering a large fraction of each reference genome in form of relatively few (and large) scaffolds.

To test how well each assembler copes with different degrees of read coverage reference datasets were created and assembled by spiking artificial reads from two reference genomes with distinct genome sizes (*Methanosarcina mazei* with 4.1 Mb and *Methanothermobacter marburgensis* with 1.6 Mb) into the KBF dataset at different coverage values (see Materials & Methods). The fraction of each spiked-in reference genome, which could be recovered at different read coverage levels was then compared for each assembler (Fig 9A & 9B, S5 Table). Based on the assumption, that a smaller genome size is correlated with lower overall complexity, better recovery rates were expected for the smaller reference genome. Generally, metaSPAdes, IDBA-UD and MegaHit show the highest sensitivity and best genome recovery rates with more than 50% of each reference genome being reconstructed already 3x read coverage. Almost complete genome reconstruction was achieved at 6x coverage. In contrast, the lowest sensitivity was displayed by Ray Meta and Omega at k-mer lengths or overlap-cutoffs of 101, respectively. In these assemblies, more than 24x coverage was required, to reconstruct approximately 50% of the reference genomes.

However, for binning purposes, not only the fraction of the reconstructed genome is of interest, but also the number and average size of the involved scaffolds. For this purpose, we defined a variable "recovery perfomance", which is analog to the above described "assembly performance" and is calculated as the product of reference genome recovery rate (in percent) and the N50 of the involved scaffolds (in kilobases). This way, the highest values are obtained by assemblers, which reconstruct the most of each reference genome in form of the fewest, but largest, scaffolds (Fig 9C & 9D). The results show that metaSPAdes achieved the by far highest recovery performance while requiring the lowest read coverage. Nonetheless, recovery rates could be further improved with increasing read coverage. This was most pronounced for the small 1.6 Mb reference genome which was completely reconstructed in only two scaffolds (one of which was more than 1.5 Mb in length) at high read coverage levels above 48x. Therefore, this assembler is particularly suited for binning and genome reconstruction of single species genomes from mixed communities. However, relatively high recovery rates were also achieved by Ray Meta at k-mer lengths 101 and 21, even though efficient recovery only began at relatively high read coverage levels (24-48x). The recovery performances of IDBA-UD and MegaHit were highest when read coverage was low. However, already at moderate coverage levels above 12x the recovery rate stopped increasing with coverage. In the case of MegaHit, recovery rates even began to deteriorate at coverage levels above 24x. A similar deteriorating effect can also be seen for MegaHits’ predecessor, SOAPdenovo2, but only at higher coverage levels of >48x. This shows that MegaHit is particularly biased towards the assembly of low abundant genome fragments.

## 3. Conclusions

The presented overview, comparison and evaluation of popular freely available short-read metagenome assembly tools will help researchers, especially microbiologists new to this field, to grasp the different working principles and individual peculiarities of each tool. Furthermore, it will help researchers to choose the most appropriate methods for answering their specific biological question and comparing already published metagenomes. Depending on the scientific goal and research question asked, different assembly tools proof optimal. In addition, we provide very detailed background information on short read processing and assembly techniques, necessary for establishing or adapting personalized metagenome assembly and analysis workflows in the S1 Appendix. To this end we also include a non-exhaustive list of helpful and informative online resources in S4 Table.

Our presented test cases reveal that the choice of assembly tools should certainly not be governed by simple scaffold size statistics alone. While large scaffolds are often desirable, a strong emphasis should also be laid on how well the diversity of the sampled community is represented in the assembly. Quite often, large scaffolds may come at the cost of reduced sensitivity. In contrast, relatively fragmented assemblies may actually represent a larger fraction of the actual sequence information contained in the original read dataset, as indicated by high total assembly lengths and read mapping rates. The presented test cases therefore provide a strictly application-related performance overview, focusing on the depth of information that is gained from the sequencing datasets and on how much diversity is captured by the assembly.

MetaSPAdes showed the overall best assembly size statistics while also capturing a relatively large fraction of the expected diversity. The usage of this tool is relatively simple and convenient, being basically identical to that of SPAdes, and largely flexible regarding the format of the input data. A drawback may be the reduced sensitivity for micro diversity. However, for the majority of metagenome research questions, accurate and representative consensus genomes of species should be more than sufficient. Despite its old age and lack of recent updates, Ray Meta remains an adequate option for assembling metagenomes, provided that the primary goal is to reconstruct genomes of the most highly represented members from relatively simple communities. This may sometimes require an enrichment of either the organisms of interest, e.g. by mesocosm growth experiments, or of the respective genomes within the sampled DNA, e.g. by SIP enrichments [76]. However, If the aim is however to analyze the general bacterial community in more depth and detail, other assemblers should be preferred over Ray Meta, since it does not capture the overall diversity of less abundant community members. Omega is an interesting option due to its ease of use and high N50 values. However, only little of the potential diversity and overall sequence information of the test datasets could be captured by this assembler. Nonetheless, string-graph based metagenome assemblers such as Omega will likely gain more and more attention in the near future, due to the increasing feasibility of using long-read sequencing technologies in metagenomics analyses [28–30]. Since Omega has been developed only fairly recently (Table 1), there might be significant improvements in future releases. MetaVelvet displayed low assembly length statistics, but the high sensitivity for sequence diversity resulting in representative taxonomic profiles justifies MetaVelvets broad dissemination over the years.

Both, IDBA-UD and Megahit achieved a good compromise between assembly performance, represented diversity and represented micro diversity, due to the multi k-mer approach, which maximizes the read information that can be incorporated into the assembly [52]. Also, both assemblers displayed a higher ability to reconstruct reference genomes from metagenomes at moderate to low read coverage. The assembly statistics and observed diversities were similar for both assemblers, making them both viable options for most metagenomes. IDBA-UD has become a popular and widely used tool, however computational requirements (S2 Table), user friendliness and available documentation (Table 1) are also important factors to take into consideration, especially for small workgroups with no established bioinformatics support. Since Megahit showed more favorable assembly cost-efficiencies than IDBA-UD and has all options clearly documented, it can be highly recommended. Furthermore, Megahit accepts several common file formats in compressed as well as uncompressed form, thereby saving disk space as well as time otherwise spent on file format conversions, while IDBA-UD does not support the most common NGS file format, fastq.

In conclusion, it can be said that the choice of assembler should depend on the data at hand and on the exact research question asked. Generally, the best assembly is performed by multi k-mer assemblers such as metaSPAdes, Megahit and IDBA-UD. If micro diversity is not a major issue, and the primary research goal is to bin and reconstruct representative bacterial genomes from a given environment, metaSPAdes should clearly be the assembler of choice. This assembler yields the best contig size statistics while capturing a high degree of community diversity, even at high complexity and low read coverage. If mico diversity is however an issue, or if the degree of captured diversity is far more important than contig lengths, then IDBA-UD or Megahit should be preferred. The sensitivity of these assemblers, both for diversity as well as micro diversity, makes them optimal choices when trying to discover novel species in complex habitats. Whenever computational resources become limiting, Megahit becomes the most attractive option, due to its good compromise between contig size statistics, captured diversity and required memory. However, the bias of Megahit towards relatively low coverage genomes may provide a disadvantage for very large datasets, leading to a suboptimal assembly of high abundant community member genomes. In such cases, Megahit may provide better results when assembling subsets of the sequencing data in a “divide and conquer” approach.

## 4. Materials and Methods

### 4.1 Sampling

A Kelp sample (KBF) was collected from a *Macrocystis pyrifera* brown algae in the Kelp Forest sampled at the Hopkins Marine Station of Stanford University (lat. 36.619; long. -121.901) in November 2014 and no specific permission was required. It was stored in artificial seawater (ASW; 0.8 M NaCl, 0.06 M Na_2_SO_4_, 0.1 M MgCl_2_ x 6 H_2_O, CaCl_2_ x 2 H_2_O, 4.6 mM NaHCO_3_, 18.5 mM KCl, 1.6 mM KBr, 0.08 mM SrCl_2_ x 6H_2_O, 0.14 mM NaF) and shipped on ice to Germany. Several 5 cm^2^ pieces were cut from the algae and the biofilm was scraped off using a sterile scalpel. Kelp pieces and scraped off biofilm were stored in fresh ASW at -20°C until further processing.

Marburg Forest soil was sampled at the Lahnberge in Germany (lat. 50.80; long. 8.812) at the sampling site of the Max Planck Institute for Terrestrial Microbiology in October 2014 and no specific permission was required. The upper 5 cm of the soil was collected taking care to exclude debris, such as leaves, and stored at -20°C until further processing. Both field studies did not involve endangered or protected species.

### 4.2 DNA extraction

**KBF:** Kelp pieces and 1.5 ml of ASW containing the biofilm were transferred to a sterile microcentrifuge tube, vortexed pulse-wise and shaken for 5 min to release some extra biofilm from the Kelp. Subsequently, Kelp pieces were removed from the suspension via gravity flow filtration using a polycarbonate filter with 10 μm pore size (Celltrics filter, Partec, Münster, Germany). Afterwards, microbial cells were harvested by centrifugation (40 min, 16,000 g, 4°C) and re-suspended in 950 μl lysis buffer (40 mM EDTA, 50 mM Tris-HCl, 0.75 M Sucrose). The cells were lysed by adding 25 μl lysozyme (final concentration of 1 mg/ml; incubation for 45 min at 37°C under slight movement), 60 μl SDS (final concentration 1 mg/ml) and 6 μl proteinase K (final concentration 0.2 mg/ml; incubation for 60 min at 55°C under slight movement). DNA was extracted from the lysate by two rounds of standard phenol-chloroform extraction using an equal volume of phenol:chloroform:isopentanol [25:24:1 (v:v:v)], followed by two chloroform extractions using an equal volume of chloroform:isopentanol [24:1 (v:v)]. Final purification and concentration was performed via alcohol precipitation using an equal volume of ice-cold isopropanol and sodium acetate (final concentration 0.3 M) by incubating overnight, followed by centrifugation (20 min, 16,000 g, 4°C) and two washing steps with ice-cold ethanol [70%]. The DNA pellet was then air-dried and re-suspended in 20 μl nuclease free water.

**MFS:** 0.5 g of soil and 1 ml of extraction buffer [1.25 g SDS, 0.2 M NaH_2_PO_4_, 0.1 M NaCl, 0.05 M EDTA, pH 8] were directly added to 200 μl of nucleic acid free glass beads in a 2 ml screw-cap tube and bead beat for 45 sec at 6 m/s (PowerSoil DNA Isolation Kit, MoBio Laboratories Inc., Carlsbad, CA, USA) [77]. The lysate was centrifuged (5 min, 19,000 g, 4°C) and the supernatant was transferred into a new 2 ml microcentrifuge tube. Phenol-chloroform and chloroform extractions were performed as for the KBF sample. In the final aqueous phase DNA was precipitated by adding 1 ml of precipitation solution [20% (w:v) PEG 6000, 2.5 M NaCl], mixing, incubation (1 h at room temperature) and centrifugation (30 min, 19,000 g, 20°C). The DNA precipitate was then washed twice with ice-cold ethanol [70%], air-dried and dissolved in 100 μl nuclease-free water. Despite the lack of a pre-filtration procedure, the influence of eukaryotic genomes in the resulting DNA was found to be minimal (Fig 1, S1 Fig). DNA purities were determined photometrically using a NanoDrop^™^ 2000 spectrometer (Thermo Fischer Scientific, Wilmington, USA) and concentrations were measured using a Qubit^™^ 1.0 fluorometer and the dsDNA HS assay kit (Life Technologies, Darmstadt, Germany).

### 4.3 Library preparation and sequencing

DNA was sheared using a Covaris S220 sonication device (Covaris Inc; Massachusetts, USA) with the following settings: 55 s, 175 W, 5% Duty factor, 200 cycles of burst, 55.5 μl. Sequencing libraries were prepared using the NEBNext Ultra^™^ DNA Library Prep Kit for Illumina (New England Biolabs, Frankfurt, Germany) as per the manufacturer’s instructions. For KBF, 5 ng sheared DNA was used and 12 cycles of enrichment PCR. For MFS, 25 ng sheared DNA was used as input and 11 cycles of enrichment PCR, due to a higher inhibitory humic acid content in this sample. The libraries were then sequenced on an Illumina MiSeq machine using v3 chemistry (600 cycles).

### 4.4 Read processing

Raw sequences were subjected to adapter clipping and quality trimming using Trimmomatic [78] with the following arguments: “LEADING:3 TRAILING:3 SLIDINGWINDOW:4:15 MINLEN:105”. Overlapping read pairs were identified and merged using FLASH [79] using the following arguments “-m 50 -r 220 -f 450 -s 100 -x 0.15 -z -t 4”.

### 4.5 Assembly

Processed reads were assembled with different assemblers. MetaSPAdes [40], IDBA-UD [52] and Megahit [51] were used with a k-mer range of 21–101 and a step size of 10. In the case of IDBA-UD, merged reads were passed using the "—long_read" argument. Paired fastq files were converted to interleaved fasta format using the supplied script fq2fa. Assembly was performed using the "—pre_correction" option, as recommended for metagenome datasets [52]. In the case of Megahit, the "sensitive" preset option was chosen for all assemblies. For each dataset and each of the de Bruijn graph assemblers MetaVelvet, Ray meta and SOAPdenovo2 [32,43,47], two separate assemblies were performed at k-mer lengths 21 and 101, in order to demonstrate the effect on sensitivity and scaffold length. Similarly, two different overlap length cutoffs were employed for the string graph assembler Omega [48]. MetaVelvet was run using 8 parallel threads. With the exception of Omega, which does not support parallelization, all other assemblers were run with 4 parallel threads or processes. The exact command line invocations used for running each assembler are given in S1 Protocol. For all subsequent comparative analyses, only scaffolds larger than 500 bp were considered.

### 4.6 Marker gene prediction

ORF calling and total protein sequence prediction was performed using prodigal [80]with "metagenome" settings. Universal marger genes were then extracted using FetchMG (www.bork.embl.de/software/mOTU/fetchMG.html). To minimize the influence of spurious marker gene predictions, only marker gene product sequences longer than 100 aa were considered. 16S and 18S rRNA sequences were extracted from the assembled scaffolds using rnammer [81]. Ribosomal gene sequences were tested for chimeras using the online tool DECIPHER [82]

### 4.7 Analyses of marker gene phylogeny

Gene product COG201, referring to preprotein translocase subunit SecY, was chosen as a representative for the 40 fetchMG protein encoding marker genes (www.bork.embl.de/software/mOTU/fetchMG.html) present in the KBF and MFS assemblies, because it occurred in all assemblies at the highest frequency (see S3 Table). Protein sequences >100 bp were aligned using the tool muscle [83] and taxonomically classified using diamond [68] and MEGAN5 [69]. 16S and 18S rRNA gene sequences were aligned and taxonomically classified using SINA [84]. Neighbor-joining phylogenies were inferred using ARB v6.0.3 [85].

### 4.8 Clustering of marker gene sequences

Nucleotide sequences of SecY-encoding as well as 16S and 18S rRNA genes were clustered using CD-HIT [86], based on different identity cutoffs. An identity cutoff of 96% was chosen for clustering of ribosomal genes, as this represents a common threshold for defining 16S rRNA OTUs [87]. Since protein-encoding genes are less conserved than rRNA genes, due to the degeneracy of the genetic code [88], a lower cutoff of 90% was chosen for *secY* genes.

### 4.9 Reference dataset generation and assembly

For an additional evaluation of the effectiveness and reliability of genome recovery by the different assemblers, the KBF dataset was spiked in with artificial Illumina sequencing reads derived from known reference genomes. The software ART [89] was used to simulate the reads with read lengths and insert sizes observed in the KBF dataset (240 bp and 400 bp, respectively). Overlapping read pairs were merged like the original dataset using Flash [79]. Two methanogenic archaea were chosen as references, since archaea were not found in the original dataset of the KBF metagenome: *Methanothermobacter marburgensis*, which has a relatively small genome of 1.6 Mb and *Methanosarcina mazei* with a genome of 4.6 Mb (NCBI accession numbers CP001710 and AE008384, respectively). The artificial reads were spiked in to create comparable reference assembly datasets representing varying read coverage levels ranging from extremely low (1x) to extremely high (>96x). For the small *M*. *marburgensis* genome, reads were added to create coverage levels of 1, 3, 6, 12, 24, 48 and 96, while *M*. *mazei* reads were added to coverage levels of 3, 6, 12, 24, 48, 96 and 192. Reads were assembled as for the original KBF dataset. The assembled scaffolds of the reference dataset were mapped to the reference genomes using BLAST+ [90] in order to determine the alignment coverage.

### 4.10 Computational resources

Sequence data processing and assembly were performed on a bioinformatics cluster, consisting of 2 TB RAM and 16 Intel(R) Xeon(R) CPU E5-4650L processors with a frequency of 2.6 GHz, eight physical and eight logical cores each, resulting in 256 available computational cores. The operating system used was Red Hat Enterprise Linux (RHEL) Server release 6.6 (Santiago). The peak RAM usage of each assembly process was monitored.

## Supporting Information

S1 Appendix A General bioinformatics background of sequence assembly. (DOC)

S2 Appendix Glossary. (DOC)

S1 Protocol Command line invocations for running the tested assemblers. (PDF)

S1 Table Development of sequencing technologies over time. (PDF)

S2 Table Assembly scaffold size statistics and computational resources. Only scaffolds >500 bp were considered. The "read mapping rate" indicates the percentage of paired, quality trimmed reads that could be mapped back onto the respective assembly. “Assembly cost” is defined as the sum of RAM usage and the wallclock time per CPU core used. “Assembly performance” is defined as the product of N50 and the read mapping rate. “Assembly cost-efficiency” is defined as the quotient of assembly performance and assembly cost.
(XLS)

S3 Table Numbers of universal single copy marker gene products encoded on the assembled contigs of the Kelp biofilm (KBF) and Marburg forest soil (MFS) samples. Marker gene products were identified by the software FetchMG (www.bork.embl.de/software/mOTU/fetchMG.html). For each marker gene and assembly, two values are given. The first value ("all") represents the total count of gene products for each marker gene. The second value ("< 90% identity") indicates the number of distinct marker gene products, after clustering of all closely related protein sequences with more than 90% amino acid sequence identity using CD-hit [75].
(XLS)

S4 Table Diversity and Micro diversity based on CD-Hit clusters of selected marker genes. Applied identity cutoffs are indicated for each marker gene and dataset. For each dataset and assembly, the number of clusters shared with other assemblies is given. "Singleton OTUs" indicate the number of OTUs consisting of only one sequence by only one assembly. "Multicopy OTUs" indicate the number of OTUs containing multiple sequences produced by the same assembly. "Diversity" is here defined as the percentage at which each assembly contributed to the total number of OTUS (by all assemblies combined). "Micro diversity" is here defined as the fraction of OTUs containing multiple sequences produced by the same assembly.
(XLS)

S5 Table Recovery of spiked-in reference genomes. "Recovery rates" indicate the fraction of the reference genome that could be recovered from the metagenome assembly by stringent BLAST alignments. "Recovery efficiency" is defined as the product of the respective recovery rate and the N50 of the involved scaffolds. Since for binning purposes fragment lengths >1 kb are of most interest, all statistics are additionally supplied based exclusively on scaffolds >1kb.
(XLS)

S6 Table Non-exhaustive list of helpful and informative weblinks. (PDF)

S1 Fig Predicted diversity and relative abundance of organisms represented in the unassembled read datasets. Taxon assignments are based on PhyloSift [71] analyses of all reads. Large ring charts show detailed breakdowns of the different phyla representing the bacterial fractions in each dataset. The proteobacterial fraction is further broken down into the represented classes of this phylum. Small pie charts indicate the relative abundances of *Bacteria*, *Eukaryota*, *Archaea* and unclassified organisms within the subset of reads which could be assigned at least to the “cellular life form” level.
(PDF)

S2 Fig Differences in phylogenetic profiles based on different assemblies of Kelp Biofilm (KBF) and Marburg forest soil (MFS) samples, on class and order level. Phylogenetic profiles are based on 40 single copy marker gene products identified with fetchMG (www.bork.embl.de/software/mOTU/fetchMG.html) and annotations based on alignments against the NCBI-nr database and the least common ancestor (LCA) method implemented by MEGAN5 [69]. The phylogenetic profiles show the number of marker gene products assigned to different taxa on class as well as order level.
(PDF)
