# Supplementary material for: Comparing and Evaluating Metagenome Assembly Tools from a Microbiologist’s Perspective - Not Only Size Matters!
Source: PLoS One. 2017 Jan 18;12(1):e0169662. doi: 10.1371/journal.pone.0169662 (PMC5242441; doi:10.1371/journal.pone.0169662)
Supplement: S1 Appendix — (DOC) [file pone.0169662.s001.doc]

# S1. A General bioinformatics background of sequence assembly

Two major approaches of sequence assembly are commonly used: Overlap based approaches such as the traditional overlap layout consensus (OLC) method or the more refined string graph, and the de Bruijn graph **(see S2 Appendix)**. Both approaches have in common that they use a data structure called a “graph” to represent all connections (edges or arcs; **see S2 Appendix**) between all basic sequence elements, e.g. reads, (called nodes or vertices; **see S2 Appendix**) extracted from the sequence dataset. Such a data structure can be imagined as a map, where each basic sequence element (node) represents a location directly connected to a set of neighboring locations via different pathways (edges). Assemblies consist of the resolution of this graph by traversing through these connections in such a way, that each element is visited in the correct order, thereby linking them together to form a contiguous sequence (contig, **see S2 Appendix**).

In traditional OLC graphs, the nodes are initially formed by the sequences of the individual reads while the edges are represented by the sequence overlaps between these reads. This rather straightforward approach was the standard method for genome assemblies for decades, since it is well suitable for longer reads of about 700 bp. OLC graphs were replaced by more refined methods with the advent of short-read NGS technologies. In the de Bruijn graph approach, edges and nodes are formed not by the reads themselves, but derived from short subsequences of defined length (k-mers, **see S2 Appendix**) into which the original read sequences are split. The string graph combines elements from the OLC graph and the de Bruijn graph. These approaches have different advantages and disadvantages, as will be explained in the following sections.

## The traditional overlap layout consensus (OLC) method

The overlap layout consensus (OLC) method is rather intuitive and consists of a three steps process. The identification of overlaps between all sequencing reads is the first and most time consuming step of the assembly process. Pairwise overlaps of variable length are identified between all reads in the dataset and represented as edges between these reads in the resulting overlap graph **(Fig S1.1A+B)**. This information is used in the next step to layout the reads into the most probable contiguous sequence stretches **(Fig S1.1C)**. In the final step the consensus sequence is determined for each contig by choosing the nucleotide, which is represented by the majority of the overlapping reads for every sequence position. Any small repetitive genome region which is shorter than the read

length, is automatically resolved this way, because the ends of reads spanning such repeats will belong to unique genome regions, allowing these reads to be unambiguously positioned during the layout step. OLC based assemblers can make best use of the complete read information, if the average read lengths are larger than most repetitive regions in the sequenced genome. Any repeat, which is larger than the maximum read length results in unresolvable ambiguous overlaps between reads originating from different parts of the genome. OLC based assemblers address this problem by masking (basically hiding, **see S2 Appendix**) reads associated with repetitive regions. This way, an ambiguous connection between reads from different genomic regions is simply ignored, breaking the assembly graph at the beginning and end of large repeats. In order to arrange and scaffold the resulting contigs, all possible contig pair combinations have to be subsequently tested using paired end read mappings. Thus, OLC based assemblers are optimally adapted to sequencing data with large read lengths, which span most repetitive regions. They usually don’t work well with short read lengths prevalent since the rise of NGS techniques. Most metagenome projects make use of high throughput short read sequencing technologies, which is one of the reasons for the popularity decline of traditional OLC-based assemblers.

| 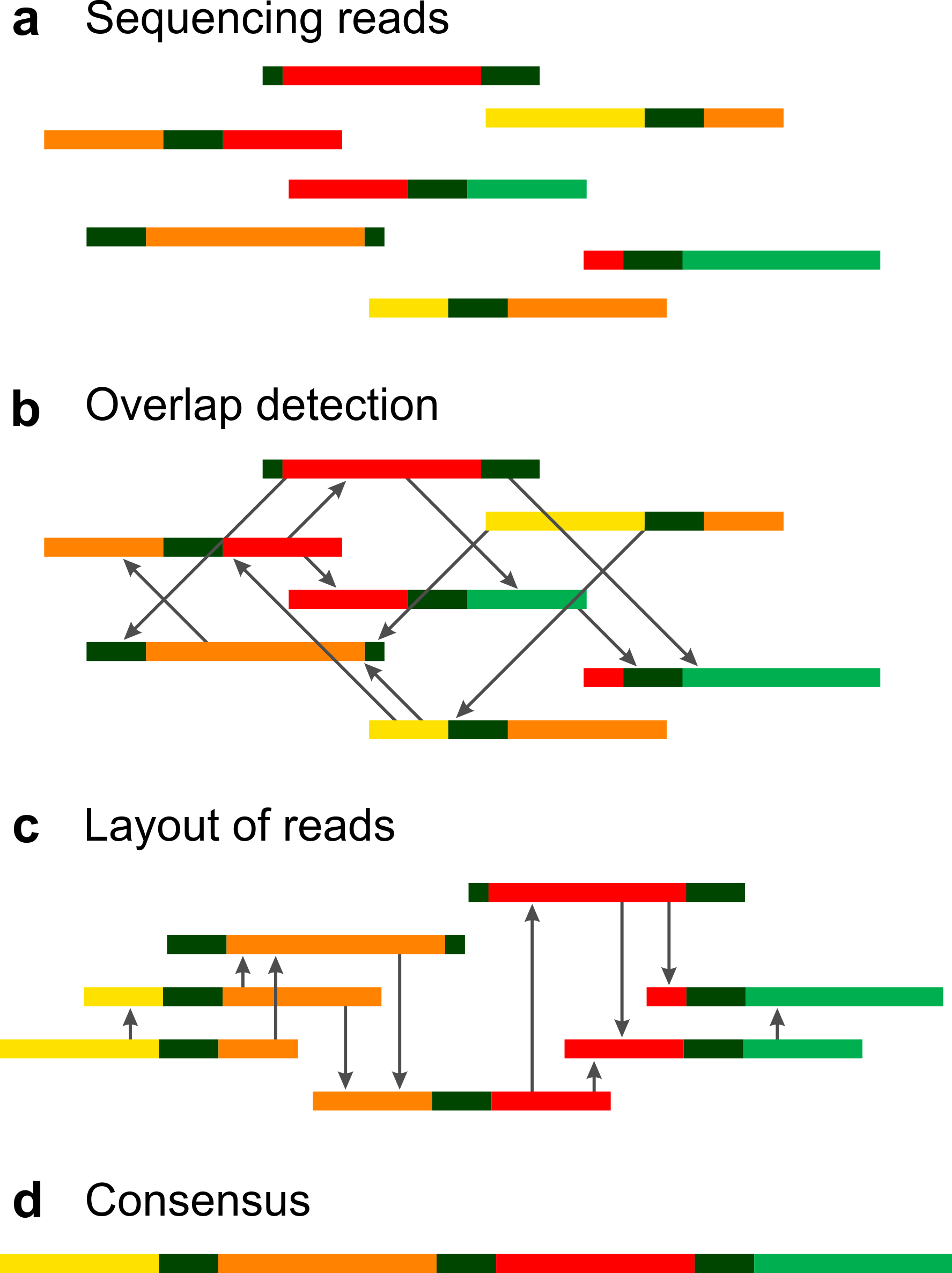 |
| --- |
| **Fig S1.1. Schematic overview of the traditional overlap layout consensus (OLC) assembly approach.** Different colors symbolize sequence stretches from different genomic regions. Grey stretches indicate small repetitive regions. **(A)** Random shotgun sequencing reads are obtained. **(B)** The read ends of all sequences are aligned to each other, in order to detect overlaps. **(C)** Even though all reads contain repetitive regions, the reads can be unambiguously placed within the layout graph, based on unambiguous averlaps at the read ends. |

## The de Bruijn graph approach

While de Bruijn graphs were originally proposed as a solution to the so-called “superstring problem” in 1946, they were first introduced for sequence fragment assembly by Idbury and Waterman [1]. The first published software tool to successfully and efficiently implement this approach

| 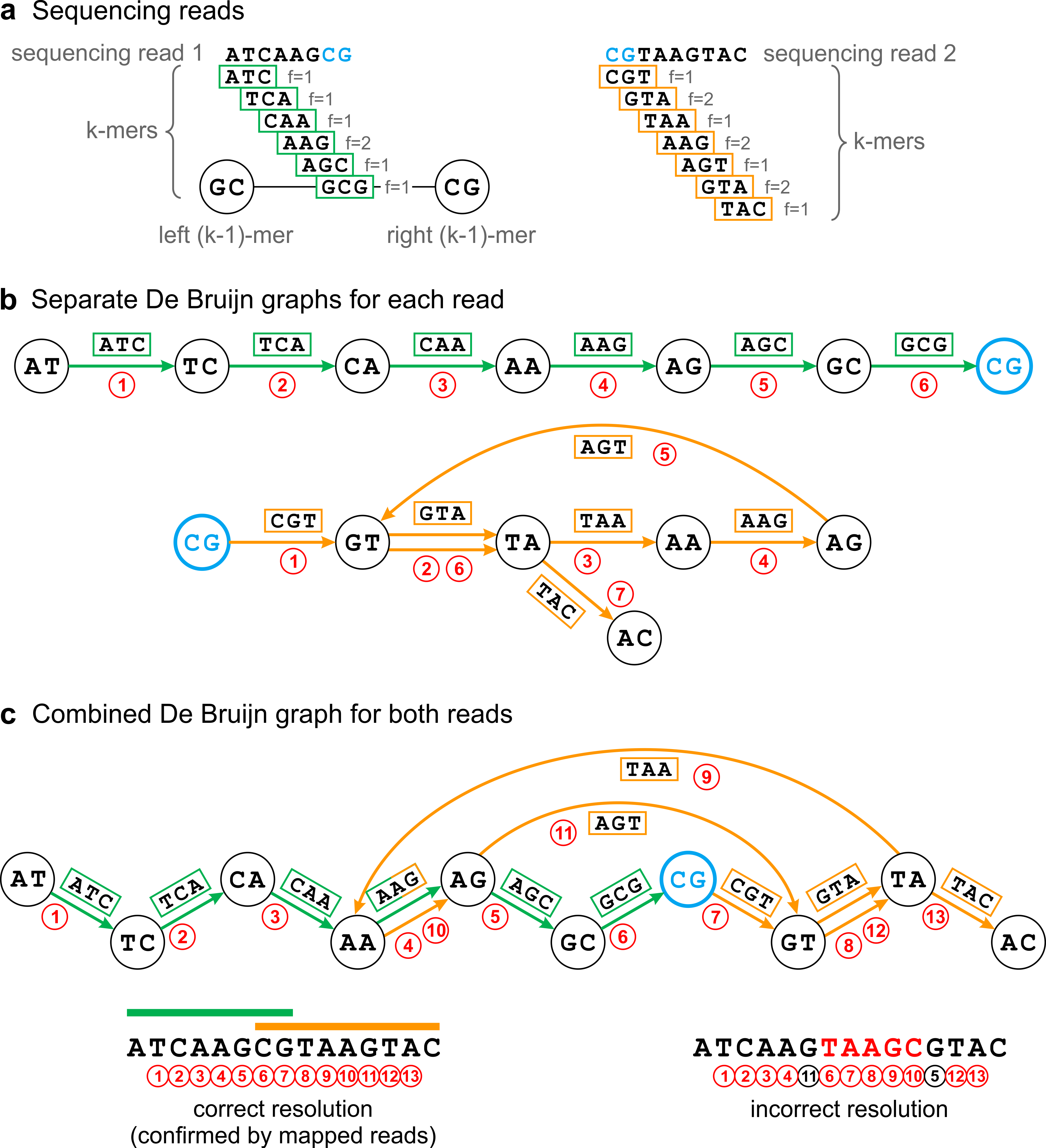 |
| --- |
| **Fig S1.2. Schematic overview of the de Bruijn graph concept.** For illustration purposes, the sequencing data is represented by only two reads, read1 and read2, which overlap by only two basepairs (marked in blue). The k-mer length is set to *k*=3. **(A)** All overlapping k-mers are derived from the reads and counted (f = frequency). Neighboring k-mers are shifted by one basepair. This means adjacent k-mers share exactly *k*-1 basepairs (k-1mers) to their left or right. On the other hand, this also means that neighboring k-mers may be identified, based on identical left or right k-1mers. **(B)** Examples of de Bruijn graphs constructed separately for each read. Each k-mer represents an edge connecting its left and right k-1mers (nodes). K-mers which are identical in their left or right k-1mer share a node in the graph, indicating that they may occur adjacent to each other. By following these connections, it is possible to reconstruct the original read sequences. Graphs are resolved by following these paths beginning at nodes with less ingoing than outgoing edges (indicating the start of a path) and ending at nodes with less outgoing than ingoing egdes (indicating the end of a path). The graph for read1 (green) forms a single straight path (visiting edges 1-6 consecutively) because all k-mers are unique within this read (although k-mer "AAG" also occurs on read2). In contrast, read2 (orange) contains a repetitive k-mer ("GTA", edges 2+6) which introduces a branched path to the graph. Nevertheless, there exists only one possible path that visits every node exactly once (visiting edges 1-7 consecutively), illustrating that repetitive regions can be resolved without breaking the de Bruijn graph. **(C)** Combined de Bruijn graph for read1 (green) and read2 (orange). An additional branch is introduced to the graph by the repetitive k-mer "AAG" occuring in both reads. As a result, the graph may be equally resolved by following different paths. However, the correct path can be identified by mapping the reads back to the graph. |

for genome assemblies was EULER [2]. The de Bruijn graph approach to sequence assembly is very different from the OLC approach: instead of tracing the connections between each individual sequencing read, the reads are split into short overlapping subsequences of a defined length *k*, so-called k-mers **(see Fig S1.2A and S2 Appendix)**. In the next step, connections are drawn between all k-mers, which overlap without mismatches in all but one (k-1) of their bases. Since this means that connected k-mers are identical in all but one of their bases, there are only 4 theoretically possible connections to each side of each k-mer (one for every nucleotide in the genetic code), considerably simplifying the task of searching for overlaps. Each k-mer can be viewed as having a left part of length k-1 (left_k-1mer) and a right part of length k-1 (right_k-1-mer), which are identical to the corresponding right and left parts of connecting (overlapping) k-mers **(Fig S1.2)**. Different descriptions of the de Bruijn graph may differ on the exact definition of which elements make up the nodes and which make up the edges. In the original de Bruijn graph implementation of EULER, these overlapping parts (k-1mers) are referred to as the nodes of the graph, while the k-mers represent the edges between these nodes. Multiple counts of the same k-mer represent multiple edges connecting the same two nodes **(Fig S1.2B+C)**. Single nodes represent repetitive regions with multiple copy numbers with multiple ingoing and outgoing edges. Together, these edges form paths through the graph, which can be traversed in order to reconstruct contigs **(Fig S1.2C)**. Conflicting paths may be resolved by mapping the sequencing reads back to the graph.

In an ideal case the result would be a single path traversing the complete graph and visiting every edge exactly once, thereby forming a single unambiguous contig. However, in reality the de Bruijn graph (even of a single genome assembly) will often be divided into multiple unconnected subgraphs due to sequencing errors and incomplete k-mer coverage.

Despite already existing for decades, de Bruijn graph based assemblers were not widely put to use until after the advent of NGS techniques. A distinct advantage of de Bruijn graphs over traditional OLC graphs lies in the way the redundancy of high coverage NGS data is handled and exploited. Multiple overlaps between different reads increase the count (frequency) of each k-mer contained in these overlaps, but not the number of nodes in the graph. New nodes are only formed when new unique k-mers are found. As a result, de Bruijn graphs scale with the size and complexity of the target genome rather than the amount and length of the input reads. Sequencing errors drastically increase the complexity of de Bruijn graphs by introducing additional “fake” k-mers and therefore spurious branches. However, erroneous k-mers result in readily recognizable structures within the graph, so-called bubbles and tips **(see Fig S1.3A+B and S2 Appendix)**. Using the multiplicity informa­tion of the k-mers, incorrect paths within these structures can be recognized, enabling a certain extent of automated error correction during the assembly step. Nevertheless, sequencing errors cause a drastic increase of memory consumption by de Bruijn graphs, as every single nucleotide substitution adds *k* new k-mers, which have to be counted and stored in the graph. Therefore, it is desirable to remove or correct sequencing errors before graph construction. At low sequencing error rates erroneous k-mers are to be expected at significantly lower frequencies than correct k-mers. Therefore, early error correction methods were based simply on coverage cutoffs by removing all k-mers/edges with low multiplicities. For single genomes with an even coverage this works well. However, in metagenomic datasets this method removes also correct k-mers originating from very low abundant community members. Dedicated metagenome assemblers therefore use revised methods for error correction and the handling of coverage variations to address this problem.

| 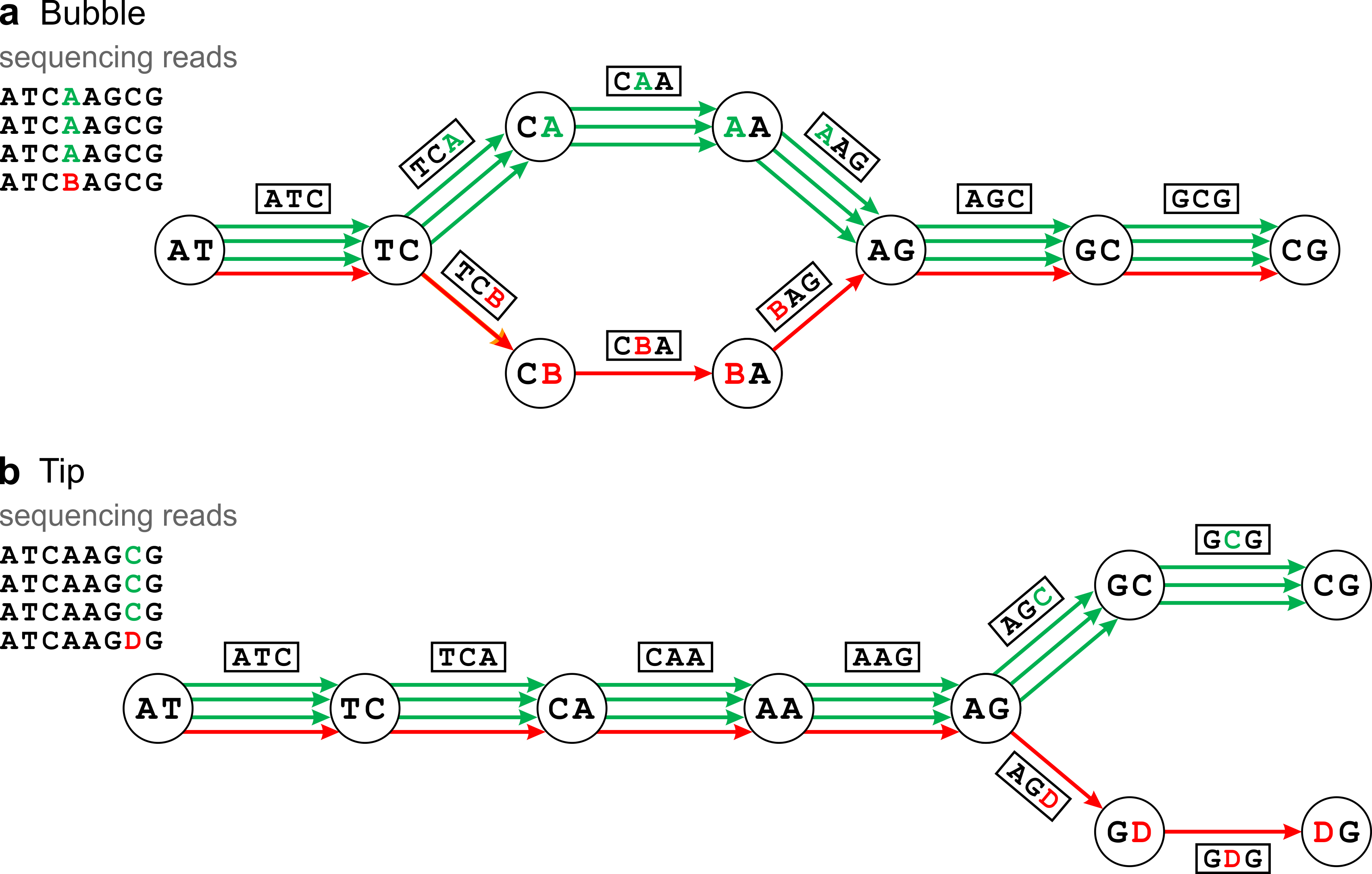 |
| --- |
| **Fig S1.3. Systematic identification and correction of single nucleotide substitution errors within de Bruijn graphs.** Correct nucleotide positions and graph edges are marked in green, incorrect nucleotides and edges in red. Erroneous reads containing a single nucleotide substitution error (marked in red) introduce *k* false k-mers to the graph, resulting in additional spurious branches. However, these form distinct structures within the graph, so-called short "bubbles" and "tips" **(see S2 Appendix**), with systematically recognizable characteristics. At low sequencing error rates, the correct path may be identified simply based on the higher number of supporting edges. **(A)** If a single nucleotide substitution error is located near the middle of a read, this k-mer will still be connected, via overlaps of length *k*-1 to correct k-mers on either side. As a result, the path through the graph will diverge at the position at which the first erroneous k-mer (= edge) occurs, and will reconverge at the position of the last erroneous k-mer. The result is a bubble like structure of the exact length *k*-1. **(B)** If a single nucleotide substitution error occurs near the beginning or end of a read, then it will only be connected to correct k-mers on one side. The other side will remain unconnected, forming a tip-like structure of the exact length *k*-1. In both cases (**A & B**) the incorrect path will be supported by significantly less edges (most likely even only one) than the correct path. Therefore, the incorrect path can automatically be recognized and removed from the graph. |

In order to take the double stranded nature of DNA and the unknown orientation of the sequencing reads into account, each k-mer is typically considered together with its reverse complement orientation. However, in the case of palindromes **(see S2 Appendix)** the forward and reverse complement orientations are identical and such k-mers can be connected in more than one orientation. This introduces additional branches to the graph and gives rise to potential inversions. Since palindromes cannot occur at uneven k-mer lengths, most de Bruijn graph assemblers (with the exception of the IDBA assemblers [3]) avoid this dilemma simply by restricting *k* to uneven numbers. Furthermore, de Bruijn graphs are not automatically broken into subgraphs whenever repeats cause ambiguously branched paths, as it would be the case for traditional OLC based assemblers. Any information on possible connections between different genomic regions remains represented in the graph and may be resolved into a single continuous path based on paired end read mappings. This makes the de Bruijn graph approach well suited for the relatively short read lengths of most NGS data, since these are less likely to span all encountered repetitive regions. The extremely high memory requirement for k-mer counting and graph construction of complex datasets, such as soil metagenomes, remains a limiting factor. Therefore, efforts for improving de Bruijn graph assemblers often resulted in reducing memory requirements, e.g. by optimizing the basic data structure and/or minimizing the influence of erroneous information by error correction.

Another major disadvantage of de Bruijn graphs is the fact that valuable context information stored within the reads is lost for assembly, because the k-mers have to be shorter than the actual read length. Theoretically possible connections between different k-mers, which are not found in the original reads, artificially increase the complexity of the graph. Also, short repeat regions, which are spanned by the original input reads but are longer than the chosen k-mer length may be more easily resolved by overlap-based assemblers. Different methods can be applied in order to retain or regain most of this context information, e.g. by mapping the input reads back to the graph. Nonetheless, sequence information from long reads may be more optimally represented by overlap-based approaches.

## The String graph approach

For the above mentioned reasons, overlap based assembly methods have been revised, by integrating key elements of the de Bruijn graph, resulting in the string graph [4]. In this approach all transitive edges **(see S2 Appendix**) are removed from the original overlap graph based on concepts originally introduced with the long read genome assembler Celera [5] ([wgs-assembler.sourceforge.net](http://wgs-assembler.sourceforge.net/)), which today is commonly used for assembling long reads (e.g. PacBio). The result is a drastically simplified and directed overlap graph. By treating overlaps between reads as nodes and the reads as edges, a string graph is derived, with basically the same properties and advantages as a de Bruijn graph, but more sequence information represented by every node and edge. However, a major disadvantage of overlap-based approaches remains, since the search for overlaps still requires alignments between every possible read combination. This makes all overlap based approaches, string graph as well as OLC, extremely time consuming for large sequencing datasets. In contrast, neighboring k-mers can only differ by one base, as they must overlap by k-1 bases. Therefore, to each side of each k-mer, there are only 4 possible sequences, which may form the required overlap (one for every nucleotide A, T, C or G). As a result, de Bruijn graph construction can be calculated relatively fast. There are several newer efficient overlap detection approaches, which have been developed to reduce the time and computational cost of overlap graph construction by using compressed data structures and read-prefix/-suffix tables for overlap detection [6,7]. Therefore, overlap based assemblers utilizing the string graph method are definitively not out of the picture for metagenomic assemblies and even experience a renaissance with the increase in read lengths of NGS technologies (e.g. PacBio, Nanopore). Recent string graph assemblers have even been designed specifically to handle short read data, such as the Omega assembler [7]. Nonetheless, the majority of currently used short read metagenome assemblers are still based on de Bruijn graphs.

## General considerations and pitfalls influencing metagenome assemblies

### Choice of k-mer

Generally speaking, shorter k-mers increase the sensitivity but reduce the specificity of an assembly, while larger k-mers have the opposite effect: The latter allow for a better resolution of small repeats but reduce the chance of encountering full overlaps of length k-1 between k-mers of overlapping reads. If the k-mer is chosen too large, minimally overlapping reads may not be connected via sufficiently overlapping k-mers, even if the reads do basically overlap and theoretically provide full coverage of the genome **(Fig S1.4A)**. Therefore, larger k-mers require a higher sequencing depth, in order to increase the likelihood of sufficiently overlapping reads at every seqeunce position. With shorter k-mers, overlaps of length >*k* between reads become more likely, reducing the need for a high sequencing depth. However, more branches are introduced into the de Bruijn graph, as overlaps between random short k-mers also become more likely and fewer repetitive regions can be resolved **(Fig S1.4B)**. A compromise between these effects can be attempted by testing several k-mer lengths and then choosing the assembly with the best combination of contig length and genome coverage statistics ([bioinformatics.net.au/software.velvetoptimiser.shtml](http://www.bioinformatics.net.au/software.velvetoptimiser.shtml)). Tools such as kmerGenie [8] and Velvet Advisor ([dna.med.monash.edu.au/~torsten/velvet_advisor](http://dna.med.monash.edu.au/~torsten/velvet_advisor/)) have been developed to predict the optimal value for k for a given read dataset, based on read length and k-mer frequencies. Other strategies may include the merging of assemblies produced with different k-mer sizes **(see S2 Appendix)** or the use of a multi k-mer assembly method as realized in the IDBA-UD assembler [3].

| 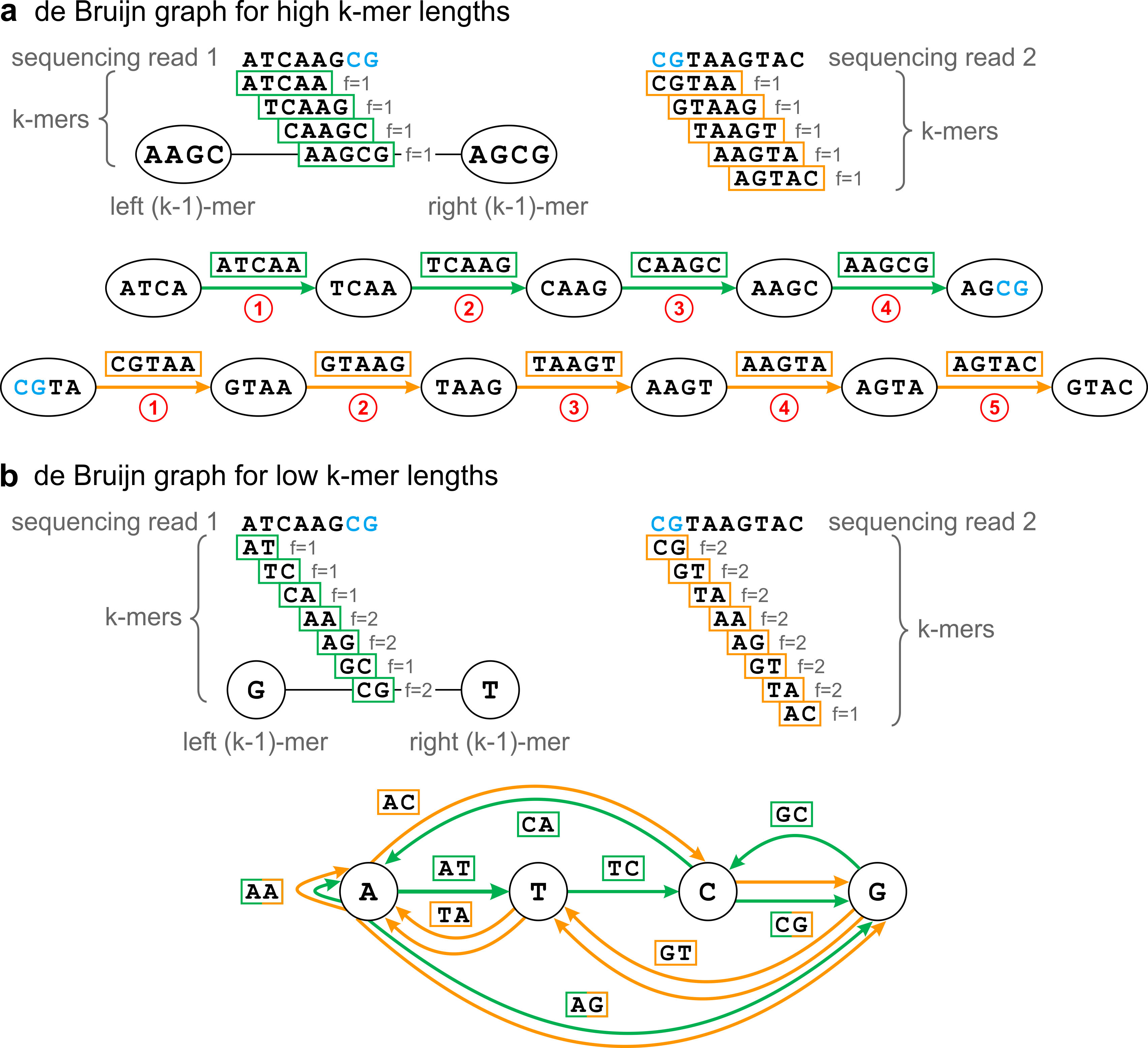 |
| --- |
| **Fig S1.4. Influence of k-mer length on assembly performance.** The same exemplary reads as in Fig S1.3 are used to illustrate the effect of varying k-mer lengths for a given read dataset. **(A)** Example for setting *k* too high (in this example *k*=5 or >60% of the read length): A high k-mer length results in more unique k-mers (with a frequency f=1). In this example, all k-mers are unique; therefore, no branches are introduced to the de Bruijn graph resulting in completely unambiguous paths. However, since the overlap between the reads (marked in blue) is shorter than the node size *k*-1, the paths of both read sequences cannot share a common node (k-1mer) and are therefore not connected. Instead, the k-mers of both reads form separate disconnected subgraphs, resulting in separate contigs. In order to connect the k-mers of both reads, an overlap of at least four base pairs would be necessary (node-length = *k*-1 = 4). Since the likelihood of such overlaps increases with read coverage, large k-mers require high coverage. **(B)** Example for setting *k* too low (in this example *k*=2 or 25 % of the read length): At low k-mer lengths more k-mers are found at higher frequencies, and fewer k-mers are unique. This results in a more branched graph. In this example, both read sequences are well connected within the graph despite the low read overlap and coverage. However, the numerous branches make it impossible to identify the most likely pathway and derive the correct contig sequence. |

### Read processing

Sequencing reads are not produced in constant quality from read start to read end. Signal intensities weaken during the course of a sequencing run due to decomposing sequencing chemistry. The trustworthiness of the base calling **(see S2 Appendix)** will therefore often deteriorate near the end of each read. This is represented in the quality values, which are estimated for every nucleotide of each read and included in the commonly used fastq sequencing file format **(see S2 Appendix)** produced by Illumina and many other NGS platforms. Deviating file formats used by some platforms, such as the SFF files produced by 454, are usually readily convertible into the common fastq format. In addition, raw sequencing reads often contain residual sequencing adapter regions or even contaminating sequences. All of these factors introduce new branches to de Bruijn graphs, making them harder to be resolved and increasing memory requirements. For optimal assembly efficiency, adapter and low quality regions should therefore be assessed and optionally trimmed from the reads. FastQC ([www.bioinformatics.babraham.ac.uk/projects/fastqc/](http://www.bioinformatics.babraham.ac.uk/projects/fastqc/)) is a popular tool used to assess adapter content and quality statistics of read datasets. Removal of adapter and low quality regions is performed by a large range of publicly available software tools, such as the FastX toolkit ([hannonlab.cshl.edu/fastx_toolkit](http://hannonlab.cshl.edu/fastx_toolkit/)), BBtools ([sourceforge.net/projects/bbtools](http://sourceforge.net/projects/bbtools/)), Trimmomatic [9], cutadapt [10] or Sickle ([github.com/najoshi/sickle](https://github.com/najoshi/sickle)) **(Table S4)**.

The most frequent contamination in Illumina datasets is the PhiX phage sequence that is often spiked into sequencing runs as a control or to ensure a balanced GC distribution. PhiX associated reads routinely turn up in the read datasets of every sample multiplexed sample. These can easily be filtered from the datasets using short read aligners such as Bowtie2 and BWA [11] or dedicated tools such as FASTQ-screen ([www.bioinformatics.babraham.ac.uk/projects/fastq_screen](http://www.bioinformatics.babraham.ac.uk/projects/fastq_screen/)).

It is also worth considering that in most cases the insert size range varies strongly enough, that a certain fraction of read pairs may display overlapping read sequences. Such reads pairs may be identified and merged into a single longer sequence using a range of software tools [12,13]. The increased merged read length enables the application of larger k-mer values and lowers the risk of "loosing" k-mers due to low coverage or weakly overlapping reads **(Fig S1.4)**, thereby improving the assembly.

### Error correction

Traditionally, any kind of error correction is deemed unsuitable for metagenome datasets. It is feared that error correction may remove micro variation from the metagenome data, because sequencing errors cannot be reliably distinguished from naturally occurring single nucleotide polymorph­isms (SNPs). However, in many cases it can be more preferable to obtain long consensus sequences for a complete species than to reconstruct micro variation within highly related substrains. If micro variations are not distinguishable from sequencing errors within metagenomes, their detection may not provide reliable insights anyway. Obviously, error correction methods, which are based on simple coverage cutoffs, can remove valuable sequencing information from less abundant species and are therefore not applicable for metagenomes. However, recent correction approaches as implemented in SPAdes, IDBA-UD, or the khmer suite [3,14,15], are restricted to low-abundant k-mers within otherwise high abundant reads and therefore remove only the most likely sequencing errors. Not only does this exclude spurious branches from the assembly graphs but also significantly reduce memory requirements during the de Bruijn graph construction.

### Digital normalization

Reducing the size and complexity of a sequencing dataset results in greater speed and lower computational cost of graph-building and assembly. A primitive way to achieve this goal is to randomly subsample the reads (e.g using seqtk; [github.com/lh3/seqtk](https://github.com/lh3/seqtk)). However, such an approach would reduce redundant and unique sequence information alike, making underrepresented genome regions harder, if not impossible, to assemble. Digital normalization, on the other hand, aims at specifically reducing the read coverage for overrepresented genomic regions while conserving low coverage ones [16,17]. The size of read datasets containing redundant information and large coverage variations can be nicely reduced this way, while minimizing the loss of valuable sequence information. Apart from simplifying assemblies, digital normalization also reduces disk storage space requirements. However, it is important to note that although most sequence information is conserved, coverage information will be partially lost. This is not a disadvantage for samples treated with Multi Displacement Amplification (MDA) [18] (such as single cell genomes) before sequencing, since the coverage information is greatly biased due to the non-uniform amplification anyway. For metagenome assembly of untreated DNA, on the other hand, this coverage information may be used for efficient graph resolution, and it's loss may lead to some repeats or cross-species homologies not being resolved correctly.

The software suites khmer, BBTools ([sourceforge.net/projects/bbmap](https://sourceforge.net/projects/bbmap/)) and NeatFreq ([github.com/bioh4x/NeatFreq](https://github.com/bioh4x/NeatFreq)) provide normalization methods based on the median k-mer frequencies of each read. By measuring median k-mer frequencies, read coverage (and redundancy) can be estimated reliably without having to align reads to reference sequences. High k-mer frequencies indicate high redundancy, and corresponding reads will only be retained up to a coverage value determined by a user-defined cutoff. Khmer and BBtools both use a memory efficient probabilistic data structure called count-min sketch [19,20] for k-mer counting, and are therefore less RAM consuming than standard de Bruijn graph assembly. Both suites also include methods to reduce sequencing errors by trimming low-abundant k-mers from otherwise highly redundant reads. NeatFreq is dependent on user supplied k-mer frequency values **(see S2 Appendix)**, but includes a pipeline for their calculation using GenomeTools’ Tallymer program [21].

### Read partitioning

Read partitioning consists of predicting groups of potentially related reads in the unassembled dataset and sorting them into separate small subsets of reduced complexity. Ideally, each subset or partition would represent just a few or even only a single species and the assembly of such subsets can be expected to require far less computational resources than the original complete dataset. The khmer suite includes partitioning methods based on graph connectivity. A preliminary assembly graph is created from digitally normalized read data using a highly memory efficient probalistic data structure, called bloom filter [22] to represent the de Bruijn graph. Genome fragments from unrelated species mostly form separate unconnected subgraphs within the assembly graph. These subgraphs are then identified and the associated reads are sorted into separate partitions. Although the complexity of each partition is greatly reduced, these partitions do not necessarily represent single species, as genome fragments from unrelated species may be connected in the same subgraph due to orthologous regions (e.g. transposons). BBtools on the other hand provides a different approach for read partitioning. Here, reads are partitioned simply based on read coverage as predicted from median k-mer frequencies. Distinct coverage variations indicate reads originating from different species. The resulting partitions greatly reduce complexity without greater loss in the sequence information. Nevertheless, different species may occur in equal abundances and therefore be represented in the same partition.

The recently described procedure of latent strain analysis (LSA; [github.com/brian-cleary/LatentStrainAnalysis](https://github.com/brian-cleary/LatentStrainAnalysis)) partitions reads based on covariance across multiple samples [23]. Since reads generated from fragments of the same origin genome can be assumed to share identical relative frequencies across samples, this method can achieve a higher species resolution than the previously mentioned read partitioning methods. Since generating multiple metagenomics datasets from almost identical samples increases the cost and labor intensiveness of metagenomics projects, such an approach would not have been commonly applicable in the past. However, due to the shrinking costs and rising efficiency of NGS techniques, sequencing and analysis of multiple related samples is no luxury anymore and has even become a prerequisite for several post-assembly binning **(see S2 Appendix)** methods [24–26].

## References

1. Idury R, Waterman M. A New Algorithm for DNA Sequence Assembly. J Comput Biol. 1995; doi:10.1089/cmb.1995.2.291

2. Pevzner PA, Tang H, Waterman MS. An Eulerian path approach to DNA fragment assembly. Proc Natl Acad Sci USA. 2001; doi:10.1073/pnas.171285098

3. Peng Y, Leung HCM, Yiu SM, Chin FYL. IDBA-UD: a de novo assembler for single-cell and metagenomic sequencing data with highly uneven depth. Bioinformatics. 2012; doi:10.1093/bioinformatics/bts174

4. Myers EW. The fragment assembly string graph. Bioinformatics. 2005; doi:10.1093/bioinformatics/bti1114

5. Myers EW, Sutton G, Delcher A, Dew I, DP F, Flanigan M, et al. A Whole-Genome Assembly of Drosophila. Science (80- ). 2000; doi:10.1126/science.287.5461.2196

6. Simpson JT, Durbin R. Efficient de novo assembly of large genomes using compressed data structures. Genome Res. 2012; doi:10.1101/gr.126953.111

7. Haider B, Ahn T-H, Bushnell B, Chai J, Copeland A, Pan C. Omega: an Overlap-graph de novo Assembler for Metagenomics. Bioinformatics. 2014; doi:10.1093/bioinformatics/btu395

8. Chikhi R, Medvedev P. Informed and automated k-mer size selection for genome assembly. Bioinformatics. 2014; doi:10.1093/bioinformatics/btt310

9. Bolger AM, Lohse M, Usadel B. Trimmomatic: a flexible trimmer for Illumina sequence data. Bioinformatics. 2014; doi:10.1093/bioinformatics/btu170

10. Martin M. Cutadapt removes adapter sequences from high-throughput sequencing reads. Embnet.journal. 2011; doi:10.14806/ej.17.1.200

11. Hatem A, Bozdağ D, Toland AE, Çatalyürek Ü V. Benchmarking short sequence mapping tools. BMC Bioinformatics. 2013; doi:10.1186/1471-2105-14-184

12. Masella AP, Bartram AK, Truszkowski JM, Brown DG, Neufeld JD. PANDAseq: paired-end assembler for illumina sequences. BMC Bioinformatics. 2012; doi:10.1186/1471-2105-13-31

13. Magoč T, Salzberg SL. FLASH: fast length adjustment of short reads to improve genome assemblies. Bioinformatics. 2011; doi:10.1093/bioinformatics/btr507

14. Bankevich A, Nurk S, Antipov D, Gurevich AA, Dvorkin M, Kulikov AS, et al. SPAdes: a new genome assembly algorithm and its applications to single-cell sequencing. J Comput Biol. 2012; doi:10.1089/cmb.2012.0021

15. Crusoe MR, Edvenson G, Fish J, Howe A, McDonald E, Nahum J, et al. The khmer software package: enabling efficient sequence analysis. 2014; doi:doi:10.6084/m9.figshare.979190

16. Brown CT, Howe A, Zhang Q, Pyrkosz AB, Brom TH. A Reference-Free Algorithm for Computational Normalization of Shotgun Sequencing Data. Available: arXiv:12034802v2. 2012; Accessed 27 January 2016.

17. McCorrison JM, Venepally P, Singh I, Fouts DE, Lasken RS, Methé BA. NeatFreq: reference-free data reduction and coverage normalization for De Novo sequence assembly. BMC Bioinformatics. 2014; doi:10.1186/s12859-014-0357-3

18. Dean FB, Hosono S, Fang L, Wu X, Faruqi AF, Bray-Ward P, et al. Comprehensive human genome amplification using multiple displacement amplification. Proc Natl Acad Sci USA. 2002; doi:10.1073/pnas.082089499

19. Cormode G, Muthukrishnan S. An improved data stream summary: the count-min sketch and its applications. J Algorithms. 2005; doi:10.1016/j.jalgor.2003.12.001

20. Crusoe MR, Edvenson G, Fish J, Howe A, McDonald E, Nahum J, et al. The khmer software package: enabling efficient sequence analysis. 2014; doi:10.6084/m9.figshare.979190

21. Kurtz S, Narechania A, Stein JC, Ware D. A new method to compute K-mer frequencies and its application to annotate large repetitive plant genomes. BMC Genomics. 2008; doi:10.1186/1471-2164-9-517

22. Pell J, Hintze A, Canino-Koning R, Howe A, Tiedje JM, Brown CT. Scaling metagenome sequence assembly with probabilistic de Bruijn graphs. Proc Natl Acad Sci USA. 2012; doi:10.1073/pnas.1121464109

23. Cleary B, Brito IL, Huang K, Gevers D, Shea T, Young S, et al. Detection of low-abundance bacterial strains in metagenomic datasets by eigengenome partitioning. Nat Biotechnol. 2015; doi:10.1038/nbt.3329

24. Kang DD, Froula J, Egan R, Wang Z. MetaBAT, an efficient tool for accurately reconstructing single genomes from complex microbial communities. PeerJ. 2015; doi:10.7717/peerj.1165

25. Strous M, Kraft B, Bisdorf R, Tegetmeyer HE. The binning of metagenomic contigs for microbial physiology of mixed cultures. Front Microbiol. 2012; doi:10.3389/fmicb.2012.00410

26. Alneberg J, Bjarnason BS, de Bruijn I, Schirmer M, Quick J, Ijaz UZ, et al. Binning metagenomic contigs by coverage and composition. Nat Methods. 2014; doi:10.1038/nmeth.3103
