# Supplementary material for: Comparing and Evaluating Metagenome Assembly Tools from a Microbiologist’s Perspective - Not Only Size Matters!
Source: PLoS One. 2017 Jan 18;12(1):e0169662. doi: 10.1371/journal.pone.0169662 (PMC5242441; doi:10.1371/journal.pone.0169662)
Supplement: S2 Appendix — (DOC) [file pone.0169662.s002.doc]

## S2 Glossary

**assembly** = Process of reconstructing larger coherent genomic fragments from sequencing reads.

**base calling** = The conversion of raw images/chromatograms into nucleotide bases.

**bin** = see *binning*

**binning** = Sorting of sequence fragments (contigs, scaffolds or raw reads) into different groups -called "*bins*"- based on similarities in coverage, sequence composition and/or co-abundance across samples. Members of each group are then often assumed to originate from the same or highly related species.

**bubble** = Structure within a de Brujin graph, indicating a potential erroneous base pair substitution near the middle of a read. Such a substitution would result in erroneous k-mers, which are nonetheless connected to the correct k-mers on either side. This creates a branched path within the de Bruijn graph, which appears as a bubble **(S1 Appendix, Fig S1.3A)**. In case of a single base pair substitution, the bubble has the length *k * 2*. Note that heterologous repeats may also appear as bubbles and should be differentiated from sequencing errors based on bubble-size and k-mer frequencies.

**chimera** = A mis-assembled contig connecting either non-adjacent portions within a genome or homologous regions between genomes of different species.

**contig** = A continuous genome fragment reconstructed by assembling single reads (derived from the word “contiguous”).

**de Bruijn graph** = Modern data structure most commonly used for NGS data with high read throughput and short read lengths. Utilizes k-mers as basic sequence elements and requires exact overlaps of defined lengths between each k-mer.

**edge/arc** = Connection between nodes (basic sequence elements) within an assembly graph. Overlaps of variable length between reads for overlap graphs, perfect overlaps of defined length between k-mers or k-1mers in de Bruijn graphs.

**fasta-format** = Simple sequence file format. Each sequence is marked by a header line containing the sequence name and description, followed by one or more lines of sequence information in one-letter code.

**fastq-format** = Slightly advanced sequence file format, for bundling sequence information together with its quality score information. Every sequence is represented by 4 lines: A header line containing a sequence identifier and description (such as its read pair affiliation), the sequence in one-letter code, a "+" symbol (optionall repeating the sequence ID) and, finally the quality score for each base represented by a special one-letter ASCII code.

**graph** = In the context of genome assembly, a data structure representing connections between basic sequence elements (reads, or k-mers).

**k-mer** = Categories of short subsequences or “words“ of length *k,* into which sequences are split during de Brujin graph-based assembly and k-mer frequency-based binning approaches.

**k-mer frequency** = Number of times a specific k-mer occurs within the sequencing dataset. Used for error correction and the resolution of repetitive regions.

**k-mer size** = Length *k* of the k-mers within a de Bruijn graph.

**L50** = Descriptive statistic measurement describing the distribution of contig lengths of an assembly. Defined as the number *n* of the largest sequences that make up at least half of the total assembly.

**N50** = Descriptive statistic measurement describing the average contig or scaffold length of an assembly, with emphasis on longer sequences. Defined as the shortest of the *n* largest sequences that together make up at least half of the total assembly length (see also L50).

**node/vertex** = Basic sequence element within an assembly graph. Reads in overlap graphs, k-mers or k-1-mers in de Bruijn graphs.

**Overlap Layout Consensus (OLC)**= Traditional method of sequence assembly by building an overlap graph, laying the reads out into the most likely order and deriving the consensus sequence from overlapping reads.

**palindrome** = A self-complimentary nucleotide sequence. Such a sequence is identical in forward and in reverse complimentary form. An example would be the short sequence "AACTAGTT".

**reads** = The single (unassembled) nucleotide sequence fragments produced by a sequencing run.

**scaffold** = A semi-continuous genome fragment (also referred to as “supercontig”) consisting of multiple contigs for which the relative orientation and approximate distance is known, but intermediate sequence regions are unknown.

**sequence masking** = “Hiding” repetitive or spurious sequence regions before alignment. This is usually done by temporarily replacing such regions with special characters, such as “N”s or lower-case letters. These regions will then not be considered in subsequent analyses.

**String graph** = A refinement of overlap graphs, incorporating elements of de Bruijn graph based methods.

**tip** = Structure within a de Bruijn graph indicating an erroneous base pair substitution at the beginning or end of a read. Such a substitution would result in erroneous k-mers, which are not connected to any correct k-mers on one side, because no identical overlaps of length *k*-1 exist. This creates a branched path within the de Bruijn graph which appears as a short dead-end or “tip” of length < *k* *2 **(S1 Appendix, Fig S1.3B)**.

**transitive edge** = Direct connections between nodes of an overlap graph which skip one or two nodes in between. For example, three reads A, B and C as nodes within a graph can be correctly arranged based on the overlaps between A to B (A-B) and between B to C (B-C). If read A is long enough, it may also allow a short direct overlap from A to C (A-C). In this case this overlap represents a transitive edge between A and C. Since this edge can also be inferred from A-B and B-C, it is unnecessary and can be ignored in order to simplify the graph.
