# Supplementary material for: Comparing and Evaluating Metagenome Assembly Tools from a Microbiologist’s Perspective - Not Only Size Matters!
Source: PLoS One. 2017 Jan 18;12(1):e0169662. doi: 10.1371/journal.pone.0169662 (PMC5242441; doi:10.1371/journal.pone.0169662)
Supplement: S1 Protocol — (PDF) [file pone.0169662.s003.pdf]

Protocol S1: Command line invocations for running the tested assemblers

The datasets:

-Each sequence dataset consisted of five gzip-compressed fastq files:

- Two "orphaned" files containing reads which have lost their respective paired-end mates during quality trimming and adapter clipping:
  → `orphaned_reads_forward.fastq.gz`
  → `orphaned_reads_reverse.fastq.gz`

-One "merged" file containing the merged sequences of read pairs which overlapped by more than 50 bp with less than 15% mismatches:
  → `merged_pairs.fastq.gz`

-Two "paired" files containing the remaining forward and reverse reads for each read pair, respectively, in identical order:
  → `paired_reads_forward.fastq.gz`
  → `paired_reads_reverse.fastq.gz`

The command line invocations for each assembler:

metaSPAdes:

-A YAML file ("input.yaml") was created with the following content:

```
[
 {
 orientation: "fr",
 type: "paired-end",
 left reads: [
 "paired_reads_forward.fastq.gz"
 ],
 right reads: [
 "paired_reads_reverse.fastq.gz"
 ],
 single reads: [
 "merged_pairs.fastq.gz",
 "paired_reads_forward.fastq.gz",
 "paired_reads_reverse.fastq.gz"
 ]
 }
]
```

-metaSPAdes was called:

→ "spades.py -k 21,31,41,51,61,71,81,91,101 -t 4 –meta –phed-offset 33 –dataset input.yaml -o assembly_results"

IDBA-UD:

-The read dataset was decompressed:

```
→ gunzip *.fastq.gz
```

- Non-merged paired forward and reverse reads were combined into a single file in interleave format and simultaneously converted into fasta format using the “fq2fa” tool supplied with IDBA-UD:

```
→ fq2fa –filter –merge paired_reads_forward.fastq paired_reads_reverse.fastq
  interleaved_read_pairs.fasta
```

-merged and orphaned reads were concatenated into a single file and also converted into fasta format:

```
→ cat merged_pairs.fastq paired_reads_forward.fastq paired_reads_reverse.fastq >
  single_reads.fastq”
→ fq2fa –filter single_reads.fastq single_reads.fasta”
```

- The assembler was called:

```
→ idba_ud -r interleaved_read_pairs.fasta -l single_reads.fasta -o assembly_results –
  mink 21 –maxk 101 –step 10 –num_threads 4
```

MegaHit:

-The assembler was called using the compressed fastq files directly as input:

```
→ megahit –k-min 21 –k-max 101 –k-step 10 -m 0.2 -t 4 -1 paired_reads_forward.fastq.gz
  -2 paired_reads_reverse.fastq.gz -r
  merged_pairs.fastq.gz,orphaned_reads_forward.fastq.gz,orphaned_reads_reverse.fastq.gz
```

MetaVelvet:

-all steps were performed using two different values for <kmer>: 21 and 101

-environment variables were set to allow the use of additional threads for metavelvet:

```
→ export OMP_NUM_THREADS=7
```

-first velveth was called:

```
→ velveth assembly_results_<kmer> <kmer> -shortPaired -fastq.gz -seperate
paired_reads_forward.fastq.gz paired_reads_reverse.fastq.gz -short
merged_pairs.fastq.gz -short  orphaned_reads_forward.fastq.gz -short
orphaned_reads_reverse.fastq.gz
```

-then velvetg was called on the velveth results:

```
→velvetg assembly_results_<kmer> -ins_length 350 -ins_length_sd 200 -exp_cov auto
```

-finally meta-velvetg was run on the velvetg results

```
→ meta-velvetg assembly_results_<kmer> -ins_length 350 -ins_length_sd 200 -scaffolding
  yes
```

Ray Meta:

-all steps were performed using two different values for <kmer>: 21 and 101

```
 → mpiexec -H localhost -np 4 Ray -k <kmer> -p paired_reads_forward.fastq.gz
paired_reads_reverse.fastq.gz -s merged_pairs.fastq.gz -s
orphaned_reads_forward.fastq.gz -s orphaned_reads_reverse.fastq.gz -o assembly_results
```

SOAPdenovo2:

-a soap configuration file ("soap2.config") was created with the following content:

```
max_rd_len=600
[LIB]
avg_ins=350
reverse_seq=0
asm_flags=3
rank=1
map_len=100
pair_num_cutoff=2
q1=paired_reads_forward.fastq.gz
q2=paired_reads_reverse.fastq.gz
q=merged_pairs.fastq.gz
q=orphaned_reads_forward.fastq.gz
q=orphaned_reads_reverse.fastq.gz
```

-when using a k-mer value of 21, "SOAPdenovo-63mer" was called:

```
 → SOAPdenovo-63mer all -o assembly_results_21 -p 4 -s soap2.config -K 21 -M 3 -d 1 -R
   -F
```

-when using a k-mer value of 101, "SOAPdenovo-127mer" was called:

```
 → SOAPdenovo-127mer all -o assembly_results_101 -p 4 -s soap2.config -K 101 -M 3 -d 1
-R -F
```

Omega:

-all steps were performed using two different overlap values for <ovlp>: 21 and 101

-The read dataset was decompressed:

```
 → gunzip *.fastq.gz
```

- Non-merged paired forward and reverse reads were combined into a single file in interleave format (in this case using the program "interleave-reads.py" supplied with the khmer suite [1]):

```
 → interleave-reads.py paired_reads_forward.fastq paired_reads_reverse.fastq >>
interleaved.fastq
```

-merged and orphaned reads were concatenated into a single file:

```
 → cat merged_pairs.fastq paired_reads_forward.fastq paired_reads_reverse.fastq >
single_reads.fastq
```

-The assembler was called:

```
→ omega -pe interleaved.fastq -se single_reads.fastq -l <ovlp>
```

References:

1. Crusoe MR, Edvenson G, Fish J, Howe A, McDonald E, Nahum J, et al. The khmer software package: enabling efficient sequence analysis. 2014; doi:10.6084/m9.figshare.979190
