# Supplementary material for: Comparing and Evaluating Metagenome Assembly Tools from a Microbiologist’s Perspective - Not Only Size Matters!
Source: PLoS One. 2017 Jan 18;12(1):e0169662. doi: 10.1371/journal.pone.0169662 (PMC5242441; doi:10.1371/journal.pone.0169662)
Supplement: S1 Table — (PDF) [file pone.0169662.s004.pdf]

S1 Table. Development of sequencing technologies over time [1–6].

Time frame	<2005	2005	2008	2010	2011	2011	2011	2011	2012	2012	2013	2013	2013	2015	Future
**Sequencing technology**	Sanger (ABIXXX) [2]	454 GS20 [3]	454 GS FLX Titanium [3]	Illumina Hiseq 1000 [4]	454 GS FLX+ [3]	Illumina Miseq [4]	Ion Torrent PGM [2]	PacBio RS [5]	Ion Torrent PGM [2]	Illumina Hiseq 2000/2500 [4]	Illumina Miseq v3 [4]	TruSeq Synthetic Long-Reads (Moleculo) [4]	PacBio RS II [5]	Illumina Hiseq 3000/ 4000 [4]	Oxford nanopore MiniON [6]
**Read length [bp]**	800-1200	100	~400	100	~700	150	50-100	5000-9000	400	100-150	300	~10,000	15,000	150	>10,000
**Technique**	PCR	Emulsion PCR ampli-fication	Emulsion PCR ampli-fication	Cluster ampli-fication	Emulsion PCR ampli-fication	Cluster ampli-fication	Emulsion PCR ampli-fication	Single molecule real-time sequencing (SMRT)	Emulsion PCR ampli-fication	Cluster ampli-fication	Cluster ampli-fication	Labelled sublibraries, cluster amplification & local pre-assembly	Single molecule real-time sequencing (SMRT)	Normali zed cluster ampli-fication	Single molecule nanopore threading
**Output per run**	96 Kb	20 Mb	~400 Mb	300 Gb	~700 Mb	2Gb	100-200 Mb	100 Mb per SMRT cell	>1Gb	600-1000 Gb	15 Gb	>600 Mb long read data per library & lane (+)	1 Gb per SMRT cell	750-1500 Gb	NA
**Costs per Mb**	$2400	$166	$12	$0.04	$9	$0.50	$0.70	$2	$1	$0.04	$0.08	<$1.4 (+)	$0.60	NA	NA
**Time per run**	12h	24h	24h	9 days	24h	20-36 h	3h	2h	4 h	4-11 days	20-36 h	~2-6 days (+)	4 h	4 days	NA
**Problems/ pitfalls**	Cloning bias for shotgun libraries	Homopoly-mer stretches	Homopoly-mer stretches	GGCxG motifs	Homopoly-mer stretches	GGCxG motifs	Homopoly-mer stretches	High random error rates	Homopoly -mer stretches	GGCxG motifs	GGCxG motifs	Local pre-assembly breaks at repeats	Quite high random error rate	GGCxG motifs	High random error rates

(+) Depends on sequencing machine and size of target genome

References:

1. El-Metwally S, Ouda O, Helmy M. Next Generation Sequencing Technologies and Challenges in Sequence Assembly. New York Heidelberg Dordrecht London: Springer; 2014. doi:10.1007/978-1-4939-0715-1
2. https://www.thermofisher.com.
3. http://www.454.com.
4. http://www.illumina.com.
5. http://www.pacb.com.
6. https://www.nanoporetech.com.
