# Supplementary material for: Comparing and Evaluating Metagenome Assembly Tools from a Microbiologist’s Perspective - Not Only Size Matters!
Source: PLoS One. 2017 Jan 18;12(1):e0169662. doi: 10.1371/journal.pone.0169662 (PMC5242441; doi:10.1371/journal.pone.0169662)
Supplement: S6 Table — (PDF) [file pone.0169662.s009.pdf]

S6 Table. Non-exhaustive list of helpful and informative weblinks

Name	Weblink	Source	Description
Living in an Ivory Basement	ivory.idyll.org	C. Titus Brown, University of California Davis	Stochastic thoughts on science, testing, and programming.
The Tree of Life	phylogenomics.blogspot.de	Jonathan A. Eisen, University of California Davis	Jonathan Eisen's Blog, The Tree of Life
Homolog.us	homolog.us		Nice blog and online knowledge-base on transcriptomics, bioinformatics and computational biology with a very useful tutorials section.
BitesizeBio	bitesizebio.com		A community driven blog, offering articles on various topics in biology and bioinformatics.
Langmead lab	langmead-lab.org/teaching-materials	Ben Langmead, John Hopkins University	Introduction to sequencing algorithms and python programming.
Bioinformatics Web	bioinformaticsweb.net	Suresh Kumar	A blog on bioinformatics.
SEQanswers	seqanswers.com	[1]	Widely used knowledge-sharing resource and one of the leading forums of the next generation community.
edX	edx.org		Online learning resource offering free MOOC courses. Includes several helpful courses on Bioinformatics.
Coursera	coursera.org		Online learning resource offering free MOOC courses. Includes several helpful courses on Bioinformatics, such as the helpful "Algorithms for DNA Sequencing" course by Ben Langmead
CAMI	cami-challenge.org/		Critical Assessment of Metagenome Interpretation. A benchmarking and comparison of metagenomic methods using standardized Mock community datasets.
MG-RAST	metagenomics.anl.gov	[2]	The MG-RAST (the Metagenomics RAST) web application server is an automated analysis platform for metagenomes. It provides quantitative insights into microbial populations based on functional and phylogenetic analyses of sequence data.
Qiime	qiime.org	[3]	QIIME (Quantitative Insights Into Microbial Ecology) is an open-source bioinformatics pipeline for performing microbiome analysis from raw DNA sequencing data.

IMG	img.jgi.doe.gov/cgi-bin/m	[4][5]	The IMG (Integrated Microbial Genomes) database is a genome browsing and annotation platform. The IMG/M extension of this system provides the comparative analysis of metagenomic data.
mothur	mothur.org	[6]	The mothur project seeks to develop a single piece of open-source, expandable software to fill the bioinformatics needs of the microbial ecology community. It is a widely used tool for the analysis of 16S rRNA gene sequences.
ARB	arb-home.de	[7]	The software package ARB represents a graphically-oriented, fully-integrated package of cooperating software tools for handling and analysis of sequence information.
Galaxy	galaxyproject.org	[8]	Galaxy is an open, web-based platform enabling the creation of scientific workflows and the integration of data to make computational biology accessible to scientists that do not have programming experience.
AMPHORA	pitgroup.org/amphoranet	[9]	AMPHORA (AutoMated Phylogenomic infeRence Application) is an automated phylogenomic inference tool that can be used for high-throughput, high-quality genome tree reconstruction and metagenomic phylotyping of etagenomic datasets based on bacterial and archaeal phylogenetic marker genes.
EMBOSS	emboss.sourceforge.net	[10]	EMBOSS (European Molecular Biology Open Software Suite) is a free Open Source software analysis package specially developed for the needs of molecular biologists. It integrates a range of currently available packages and tools for sequence analysis into a seamless whole.
Bio-Linux 8	environmentalomics.org/bio-linux	[11]	A Linux distribution with an extensive repository of bioinformatic tools. Possibly discontinued within 2016. Future unsure.
BioPerl	bioperl.org	[12]	BioPerl is a collection of Perl modules for bioinformatics applications
Biopython	biopython.org	[13]	Biopython is a set of Python modules for bioinformatics applications.
BioJava	biojava.org	[14]	BioJava is a set of Java modules for bioinformatics applications

1. Li J-W, Schmieder R, Ward RM, Delenick J, Olivares EC, Mittelman D. SEQanswers: an open access community for collaboratively decoding genomes. Bioinformatics. 2012; doi:10.1093/bioinformatics/bts128

2. Meyer F, Paarmann D, D'Souza M, Olson R, Glass EM, Kubal M, et al. The metagenomics RAST server - a public resource for the automatic phylogenetic and functional analysis of metagenomes. BMC Bioinformatics. 2008; doi:10.1186/1471-2105-9-386

3. Caporaso JG, Kuczynski J, Stombaugh J, Bittinger K, Bushman FD, Costello EK, et al. QIIME allows analysis of high-throughput community sequencing data. Nat Methods. 2010; doi:10.1038/nmeth.f.303

4. Markowitz VM, Chen I-MA, Palaniappan K, Chu K, Szeto E, Pillay M, et al. IMG 4 version of the integrated microbial genomes comparative analysis system. Nucleic Acids Res. 2014; doi:10.1093/nar/gkt963

5. Markowitz VM, Chen I-MA, Chu K, Szeto E, Palaniappan K, Pillay M, et al. IMG/M 4 version of the integrated metagenome comparative analysis system. Nucleic Acids Res. 2014; doi:10.1093/nar/gkt919

6. Schloss PD, Westcott SL, Ryabin T, Hall JR, Hartmann M, Hollister EB, et al. Introducing mothur: open-source, platform-independent, community-supported software for describing and comparing microbial communities. Appl Env Microbiol. 2009; doi:10.1128/AEM.01541-09

7. Ludwig W, Strunk O, Westram R, Richter L, Meier H, Yadhukumar, et al. ARB: a software environment for sequence data. Nucleic Acids Res. 2004; doi:10.1093/nar/gkh293

8. Giardine B, Riemer C, Hardison RC, Burhans R, Elnitski L, Shah P, et al. Galaxy: a platform for interactive large-scale genome analysis. Genome Res. 2005; doi:10.1101/gr.4086505

9. Kerepesi C, Bánky D, Grolmusz V. AmphoraNet: the webserver implementation of the AMPHORA2 metagenomic workflow suite. Gene. 2014; doi:10.1016/j.gene.2013.10.015

10. Rice P, Longden I, Bleasby A. EMBOSS: The European Molecular Biology Open Software Suite. Trends Genet. 2000; doi:10.1016/S0168-9525(00)02024-2

11. Field D, Tiwari B, Booth T, Houten S, Swan D, Bertrand N, et al. Open software for biologists: from famine to feast. Nat Biotechnol. 2006; doi:10.1038/nbt0706-801

12. Stajich JE, Block D, Boulez K, Brenner SE, Chervitz SA, Dagdigian C, et al. The Bioperl toolkit: Perl modules for the life sciences. Genome Res. 2002; doi:10.1101/gr.361602

13. Cock PJA, Antao T, Chang JT, Chapman BA, Cox CJ, Dalke A, et al. Biopython: freely available Python tools for computational molecular biology and bioinformatics. Bioinformatics. 2009; doi:10.1093/bioinformatics/btp163

14. Holland RCG, Down TA, Pocock M, Prlić A, Huen D, James K, et al. BioJava: an open-source framework for bioinformatics. Bioinformatics. 2008;24: 2096–7. doi:10.1093/bioinformatics/btn397
