# Supplementary material for: Comparing and Evaluating Metagenome Assembly Tools from a Microbiologist’s Perspective - Not Only Size Matters!
Source: PLoS One. 2017 Jan 18;12(1):e0169662. doi: 10.1371/journal.pone.0169662 (PMC5242441; doi:10.1371/journal.pone.0169662)
Supplement: S1 Fig — Taxon assignments are based on PhyloSift [71] analyses of all reads. Large ring charts show detailed breakdowns of the different phyla representing the bacterial fractions in each dataset. The proteobacterial fraction is further broken down into the represented classes of this phylum. Small pie charts indicate the relative abundances of Bacteria, Eukaryota, Archaea and unclassified organisms within the subset of reads which could be assigned at least to the “cellular life form” level. (PDF) [file pone.0169662.s010.pdf]

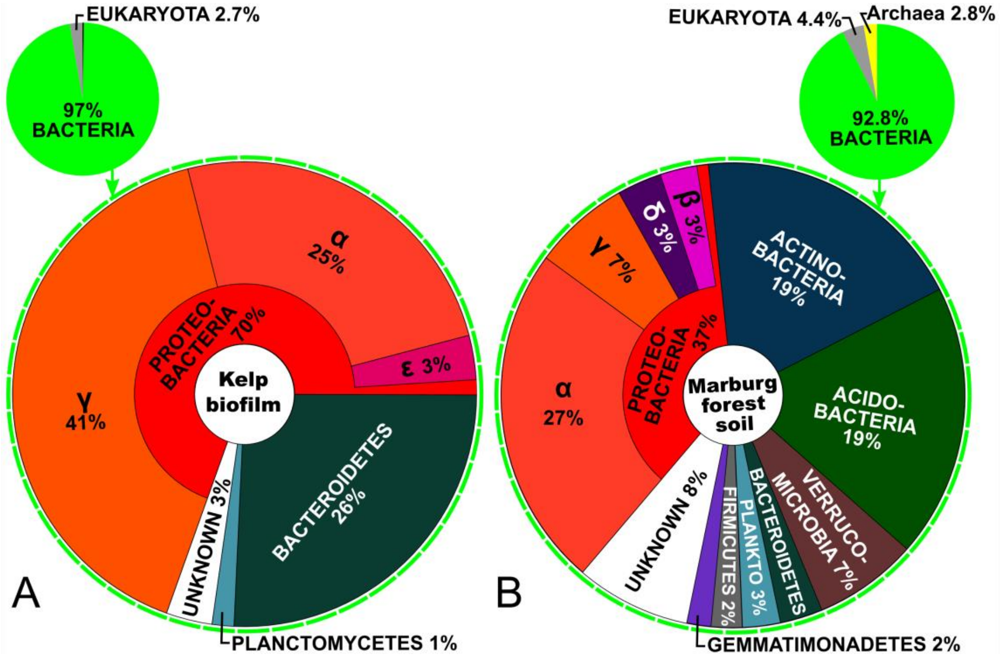

S1 Figure: Predicted diversity and relative abundance of organisms represented in the unassembled read datasets. Taxon assignments are based on PhyloSift [1] analyses of all reads. Large ring charts show detailed breakdowns of the different phyla representing the bacterial fractions in each dataset. The proteobacterial fraction is further broken down into the represented classes of this phylum. Small pie charts indicate the relative abundances of *Bacteria*, *Eukaryota*, *Archaea* and unclassified organisms within the subset of reads which could be assigned at least to the "cellular life form" level.

[1] Darling AE, Jospin G, Lowe E, Matsen FA, Bik HM, Eisen JA. PhyloSift: phylogenetic analysis of genomes and metagenomes. PeerJ. 2014; doi:10.7717/peerj.243
