# Supplementary material for: Comparing and Evaluating Metagenome Assembly Tools from a Microbiologist’s Perspective - Not Only Size Matters!
Source: PLoS One. 2017 Jan 18;12(1):e0169662. doi: 10.1371/journal.pone.0169662 (PMC5242441; doi:10.1371/journal.pone.0169662)
Supplement: S2 Fig — Phylogenetic profiles are based on 40 single copy marker gene products identified with fetchMG (www.bork.embl.de/software/mOTU/fetchMG.html) and annotations based on alignments against the NCBI-nr database and the least common ancestor (LCA) method implemented by MEGAN5 [69]. The phylogenetic profiles show the number of marker gene products assigned to different taxa on class as well as order level. (PDF) [file pone.0169662.s011.pdf]

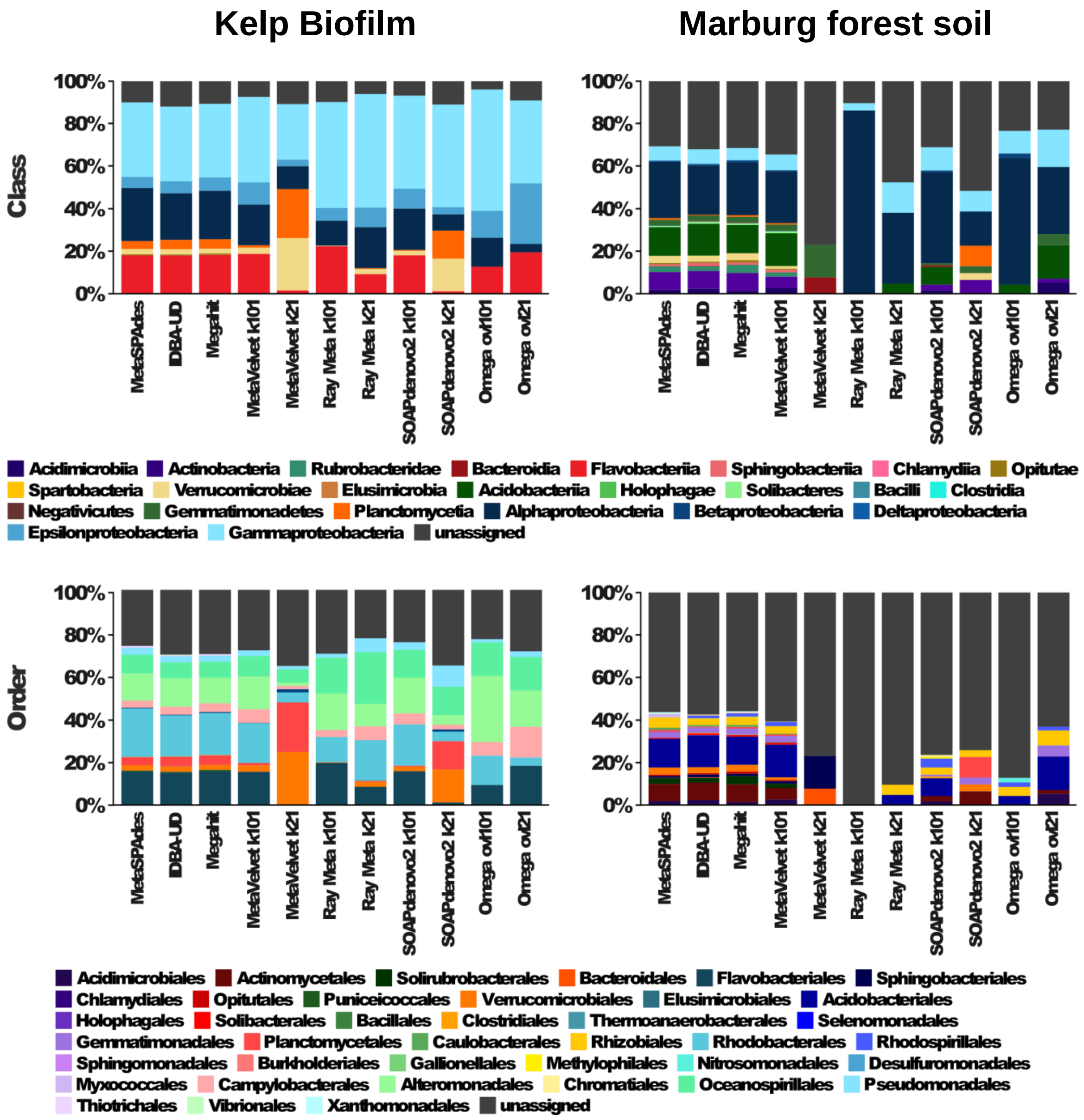

S2 Figure. Differences in phylogenetic profiles based on different assemblies of Kelp Biofilm (KBF) and Marburg forest soil (MFS) samples, on class and order level. Phylogenetic profiles are based on 40 single copy marker gene products identified with fetchMG (www.bork.embl.de/software/mOTU/fetchMG.html) and annotations based on alignments against the NCBI-nr database and the least common ancestor (LCA) method implemented by MEGAN5 [1]. The phylogenetic profiles show the number of marker gene products assigned to different taxa on class as well as order level.

[1] Huson DH, Weber N. Microbial community analysis using MEGAN. Methods Enzym. 2013; doi:10.1016/B978-0-12-407863-5.00021-6
